# Study on the Effects of Wettability and Pressure in Shale Matrix Nanopore Imbibition during Shut-in Process by Molecular Dynamics Simulations

**DOI:** 10.3390/molecules29051112

**Published:** 2024-03-01

**Authors:** Wen Jiang, Weifeng Lv, Ninghong Jia, Xiaoqing Lu, Lu Wang, Kai Wang, Yuhao Mei

**Affiliations:** 1 College of Engineering Science, University of Chinese Academy of Sciences, Beijing 100049, China; jiangwen21@mails.ucas.ac.cn (W.J.); wangkaiz@petrochina.com.cn (K.W.); meiyuhao22@mails.ucas.ac.cn (Y.M.); 2 Institute of Porous Flow and Fluid Mechanics, University of Chinese Academy of Sciences, Langfang 065007, China; 3 Research Institute of Petroleum Exploration & Development, PetroChina, Beijing 100083, China; jianinghong@petrochina.com; 4 State Key Laboratory of Enhanced Oil and Gas Recovery, Beijing 100083, China; 5 School of Materials Science and Engineering, China University of Petroleum, Qingdao 266580, China; luxq@upc.edu.cn (X.L.); s21090046@s.upc.edu.cn (L.W.)

**Keywords:** imbibition, shut-in pressure, wettability, micro-mechanism, molecular dynamics simulation

## Abstract

Shut-in after fracturing is generally adopted for wells in shale oil reservoirs, and imbibition occurring in matrix nanopores has been proven as an effective way to improve recovery. In this research, a molecular dynamics (MD) simulation was used to investigate the effects of wettability and pressure on nanopore imbibition during shut-in for a typical shale reservoir, Jimsar. The results indicate that the microscopic advancement mechanism of the imbibition front is the competitive adsorption between “interfacial water molecules” at the imbibition front and “adsorbed oil molecules” on the pore wall. The essence of spontaneous imbibition involves the adsorption and aggregation of water molecules onto the hydroxyl groups on the pore wall. The flow characteristics of shale oil suggest that the overall push of the injected water to the oil phase is the main reason for the displacement of adsorbed oil molecules. Thus, shale oil, especially the heavy hydrocarbon component in the adsorbed layer, tends to slip on the walls. However, the weak slip ability of heavy components on the wall surface is an important reason that restricts the displacement efficiency of shale oil during spontaneous imbibition. The effectiveness of spontaneous imbibition is strongly dependent on the hydrophilicity of the matrix pore’s wall. The better hydrophilicity of the matrix pore wall facilitates higher levels of adsorption and accumulation of water molecules on the pore wall and requires less time for “interfacial water molecules” to compete with adsorbed oil molecules. During the forced imbibition process, the pressure difference acts on both the bulk oil and the boundary adsorption oil, but mainly on the bulk oil, which leads to the occurrence of wetting hysteresis. Meanwhile, shale oil still existing in the pore always maintains a good, stratified adsorption structure. Because of the wetting hysteresis phenomenon, as the pressure difference increases, the imbibition effect gradually increases, but the actual capillary pressure gradually decreases and there is a loss in the imbibition velocity relative to the theoretical value. Simultaneously, the decline in hydrophilicity further weakens the synergistic effect on the imbibition of the pressure difference because of the more pronounced wetting hysteresis. Thus, selecting an appropriate well pressure enables cost savings and maximizes the utilization of the formation’s natural power for enhanced oil recovery (EOR).

## 1. Introduction

Shale reservoirs exhibit high tightness, low permeability, heterogeneity, and extensive nanopore development [1]. Conventional development techniques typically suffer from inefficiency. However, the utilization of multi-stage horizontal well volume fracturing technology for production purposes represents an effective approach [2,3]. The creation of stimulated fracture networks through hydraulic fracturing enhances the oil and gas seepage capacity of shale reservoirs. The oil present in the fractures formed during hydraulic fracturing can be extracted by utilizing the original formation pressure. Additionally, water imbibition into the matrix pores can displace the shale oil present in the nanopores, enabling its extraction to the surface and facilitating EOR [4]. After the fracturing, it is common practice to shut the well for a certain period to allow for the full manifestation of the imbibition effect.

Spontaneous imbibition refers to the process in which porous media imbibe a wetting fluid and displace the original non-wetting fluid [5,6]. In low-permeability tight oil reservoirs, capillary imbibition serves as a significant mechanism for EOR, with capillary force acting as the primary driving force for spontaneous imbibition oil recovery [7]. Numerous scholars have conducted extensive experimental studies on the mechanism of imbibition oil recovery. The findings reveal that spontaneous imbibition within the shale matrix is influenced by various factors, including reservoir fluid, reservoir rock, and physical parameters such as pore structure, pore size distribution, oil–water interfacial tension, solid-liquid interface interaction, emulsification, wettability, temperature, shale oil viscosity, initial water saturation, and permeability, among others [8,9,10,11,12,13]. However, the wettability of the rock wall plays a crucial role in determining the effectiveness of spontaneous imbibition oil recovery. A more hydrophilic core leads to a faster spontaneous imbibition velocity and higher recovery. Therefore, surfactants are frequently employed to improve the wettability of oil-wet reservoirs with limited imbibition oil recovery effects [14,15,16]. Secondly, due to the high rock fracture pressure associated with hydraulic fracturing, the pressure within fractures after fracturing is typically higher than the pressure within the matrix pores. As a result, both spontaneous imbibition within shale matrix pores and forced imbibition under the influence of high shut-in pressure take place. Thus, the shut-in pressure also serves as a critical factor that impacts the imbibition for EOR [17]. While traditional experimental methods can assess the imbibition oil recovery effect under various influencing factors, they have limitations in evaluating the oil recovery effects solely by calculating parameter changes from a macro perspective. Experimental research methods targeting various influencing factors include traditional experimental techniques such as weighing and volume observation methods, as well as advanced technologies, such as nuclear magnetic resonance (NMR) and computed tomography (CT) technology [18,19,20,21]. Such experimental methods fail to directly depict the evolving characteristics of the oil and water phases within the pores and fall short of elucidating the microscopic mechanism underlying the imbibition oil displacement process.

With the rapid advancement of computer technology, MD simulation methods have gained widespread utilization in the field of oil and gas seepage [22,23]. MD simulation starts from the atomic and molecular scale and employs classical Newtonian mechanics to describe the movement of atoms, thereby capturing the dynamic characteristics of the modeled system [24]. The MD method has reached a relatively mature stage, characterized by minimal artificial influences during the simulation process and high credibility of the obtained results. Consequently, it has emerged as a crucial tool for unraveling the intricate mechanisms governing nanoscale fluid migration. Wang et al. [25] employed MD simulations to investigate the impact of nanoparticles on spontaneous water uptake in highly confined channels. They proposed a competitive mechanism for the spontaneous imbibition of nanofluids in capillaries by integrating the dynamic process of spontaneous imbibition, the contact angle of water in the capillary tube, and the relationship between displacement and time. Similarly, Yang et al. [26] conducted MD simulations to study the imbibition of octane and water into graphite and quartz nanopores. Their findings demonstrated that oil can be imbibed into the dense organic nanopores of rocks at a faster rate than predicted by the classical imbibition model (Handy model). In another study, Nabin Kumar Karna et al. [27] performed large-scale atomistic simulations to investigate the capillary water imbibition phenomenon in pore silica nanochannels with varying heights (4–18 nm). They proposed an expression for the evolution of the dynamic contact angle in nanochannels over time, which, when combined with the Bosanquite equation, effectively explained the initial capillary rise. Additionally, Sang et al. [28] examined the imbibition of hydrocarbons in nano-kerogen pores. However, the aforementioned MD simulation studies on imbibition mainly focused on the imbibition of the wetting phase. They did not consider the displacement of the non-wetting phase from the pores. The two-phase imbibition mechanism, involving both the wetting and non-wetting phases, is more complex compared to the sole uptake of the wetting phase. Consequently, subsequent researchers conducted MD studies on the two-phase imbibition of oil and water, exploring various aspects such as the impact of polar components of shale oil on spontaneous imbibition [29] and the microscopic mechanism of water injection huff and puff for EOR [30]. Although some progress has been made, there is still limited research available on imbibition oil displacement, and the microscopic mechanism of the imbibition process involving both oil and water phases remains an area of active exploration.

In this study, we employed MD simulation to investigate the microscopic mechanism of imbibition oil displacement in matrix shale pores with different hydrophilicity and under different shut-in pressures during shut-in for the shale reservoir. Initially, we visualized and quantitatively analyzed the spontaneous imbibition process to elucidate the microscopic advancement mechanism of the imbibition front and transportation characteristics of shale oil in different hydrophilic systems. Next, we analyzed the fluid migration characteristics during the forced imbibition process in different hydrophilic systems. Subsequently, based on the simulation results and certain theoretical derivations, we evaluated the synergistic effect of pressure difference and wettability on imbibition oil displacement.

## 2. Results

### 2.1. Shale Oil Migration in Different Hydrophilic Nanopores during Spontaneous Imbibition

The spontaneous imbibition systems consisting of quartz walls W_A_ and W_B_, along with oil–water, are named the spontaneous imbibition system I (SI I) and spontaneous imbibition system II (SI II), respectively. Based on the static test results, symbols I–II represent the strongly hydrophilic system and weakly hydrophilic system, respectively. During the spontaneous imbibition process, the fluid pressure surrounding the shale matrix is in equilibrium with the original matrix pore pressure [31]. Therefore, the pressure exerted on both the left and right He plates is maintained at 40 MPa.

Firstly, MD simulations were performed on two spontaneous imbibition systems (SI I and SI II) for a duration of 1 ns. To intuitively reflect the behavior of oil–water phases in different hydrophilic systems during spontaneous imbibition, we calculated and presented the dynamic contact angle (*θ_d_*) and simulated snapshots of SI I and SI II in Figure 1. Given the asynchronous advancement of the imbibition front on the upper and lower pore walls, we determine the *θ_d_* as the arithmetic mean of the contact angles (*θ_u_*) on the upper pore wall and (*θ_l_*) on the lower pore wall (the inset in Figure 1b). As shown in Figure 1a, as the wall wettability changes from strongly hydrophilic to weakly hydrophilic, the advancement distance of the imbibition front decreases significantly. Initially, water molecules located at the entrance penetrate the pore in the form of a meniscus. The progressive movement of the meniscus compels water molecules to continuously occupy the nanopore space, leading to the displacement of the oil phase. As shown in Figure 1b, the dynamic contact angle in the strongly hydrophilic system is smaller than that in the weakly hydrophilic system, and the *θ_d_* in SI I and SI II are both less than 90°, which is in agreement with the results obtained from the static contact angle tests (Figure S5d).

To intuitively reflect the axial migration behavior of the oil phase, we calculated the velocity distribution of the oil phase along the Z-axis and the two-dimensional (2-D) density distribution of the oil phase corresponding to the simulated snapshots of the SI I and SI II at 0, 500, and 1000 ps, as shown in Figure 2. The experimental test results show that the viscosity of crude oil decreases with increasing temperature. According to test results provided by the Xinjiang Research Institute of Petroleum Exploration and Development, Jimsar’s low sweet spot shale oil exhibits a high viscosity of tens to hundreds of mPa·s. However, the corresponding experimental data shows that, at the reservoir temperature (315 K), the viscosity of Jimsar shale oil has a low viscosity close to that of water [32,33]. Therefore, it can be assumed that the viscosity of shale oil under the reservoir conditions is equal to that of water. Furthermore, assuming the fluid density and viscosity do not vary spatially, the steady-state velocity profile for an incompressible laminar fluid confined between two quartz walls is parabolic and described by the classical Hagen–Poiseuille (H–P) equation [34].

(1) $$v=-\frac{\Delta P}{2\eta }\left(z^{2}-\frac{w^{2}}{4}\right)$$

where Δ*P* is the pressure gradient along the flow direction (the pressure gradient Δ*P* during spontaneous imbibition is provided by capillary pressure); *w* and *η* are the pore width and fluid (shale oil and water) viscosity, respectively. Continuous hydrodynamics always assumes the streaming velocity of fluid vanishes at the interface. However, recent studies have confirmed that pure hydrocarbons, such as octane and decane, exhibit a slip flow behavior when flowing on hydrophilic walls [34,35]. The slip length (*L_S_*) is defined as the extrapolation distance to the surface location where the fluid velocity is equal to zero [36].

(2) $$L_{S}=\pm \frac{v\left(z_{\mathrm{surf}}\right)}{{\left(\frac{dv}{dz}\right)}_{z_{\mathrm{surf}}}}$$

where $v\left(z_{\mathrm{surf}}\right)$ is the velocity of the fluid at the position of the fluid–solid interface (*z*_surf_). ${\left(\frac{dv}{dz}\right)}_{z_{\mathrm{surf}}}$ is the velocity gradient of the fluid at the fluid–solid interface.

Therefore, taking into account the effect of interface slip, the modified velocity distribution for the H–P equation is as follows:

(3) $$v=-\frac{\Delta P}{2\eta }\left(z^{2}-\frac{w^{2}}{4}-wL_{s}\right)$$

Therefore, the *L_S_* can be obtained by fitting the velocity distribution of shale oil in Figure 2a,b using Equation (3). Before extracting the velocity distribution curve, the Gibbs dividing surface (GDS) method was employed to determine the fluid–solid interface [37]. However, considering that the imbibition front advances asynchronously on the upper and lower walls, for the accuracy and validity of the results, the velocity data on both sides of the center of the pore were fitted separately. Then the *L_S_* is equal to the arithmetic mean of the *L_S_* on the upper and lower walls of the pore. As shown in the inset in Figure 2a, the position where *z* = 0 represents the center of the pore.

From the velocity distribution diagram in Figure 2a,b, it can be observed that the velocity distribution matches well with Equation (3), and the oil phase velocity near the pore wall is much smaller. The occurrence of the slip phenomenon in the oil phase during the migration process proves that the overall push of the injected water to the oil phase is the main reason for the displacement of adsorbed oil molecules and that the oil phase has a larger *L_S_* in more hydrophilic pores. The reason for the slip is that the strong attraction between hydrocarbons and quartz walls leads to the inability of water molecules to effectively peel off the oil phase. As shown in Figure 2c, the shale oil still existing in the pores of both a strongly hydrophilic system and a weakly hydrophilic system always maintains good, layered adsorption characteristics during spontaneous imbibition. Although the methyl and hydroxyl groups of the pore wall in the weakly hydrophilic system are uniformly arranged, the different forces of the methyl and hydroxyl groups on the hydrocarbons lead to the bending of the hydrocarbons that should be adsorbed parallel to the wall. However, the adsorbed oil in the pore walls of the weakly hydrophilic system has a higher adsorption strength (Figure S1), and the bending of the adsorbed hydrocarbons does not affect the final simulation results. To further elucidate the flow behavior of shale oil during spontaneous imbibition, the mass density distribution of shale oil and the number density distribution of different hydrocarbon components after 3 ns of structural optimization, and the molecular number changes of different hydrocarbon components over time in the pores during spontaneous imbibition were calculated in Figure 3. It can be found, in agreement with the 2-D density distributions, that a distinct aggregation of hydrocarbon components on both sides of the pore wall occurs (Figure 3a,d). The oil in the pore contains three distinct adsorption layers, each with a width of about 5 Å. Figure 3a,d shows that the average bulk density of shale oil calculated by MD simulation from *z* = −11 Å to *z* = 11 Å was 0.76 and 0.758 g/cm^3^, respectively, while the density measured by the experiment was 0.81 g/cm^3^ [32]. This was because C_20_ was applied in the MD model to uniformly represent the C_20+_ molecules of the actual shale oil components, resulting in a lower density calculated by the model than that measured by the experiment. The oil within 5 Å of the quartz wall surface is defined as the first adsorption layer. The second and third adsorption layers are defined by analogy. As shown in Figure 3b,e, the heavier hydrocarbon components generally have higher adsorption density in the first adsorption layer. There is no obvious peak density of C_4_ in the first adsorption layer in the weakly hydrophilic system II because the pore wall modified by methyl groups of the weakly hydrophilic system has a stronger adsorption effect on hydrocarbons. Therefore, the pore walls of the weakly hydrophilic system have a stronger adsorption effect on heavy and medium components, leading to a further reduction in the amount of C_4_ adsorbed near the pore wall, which is a free state almost throughout the pore channels. During the imbibition period, the number of different hydrocarbon components in the first adsorption layer gradually decreases, and the number of hydrocarbons in the pores with more hydrophilic walls decreases faster (Figure 3c,f).

However, it is less clear whether the reduction in the number of different hydrocarbon components in the first adsorption layer is achieved by slipping to the outside of the pores or by desorption from the pore walls into the bulk phase fluid. To evaluate the transport characteristics of different hydrocarbon components of adsorbed oil in the pores, the migration trajectories of C_4_, C_8_, and C_20_ in the first adsorption layer after structural optimization during spontaneous imbibition are labeled as shown in Figure 4. The migration trajectories indicate that the heavy mass components in the adsorbed layer after structural optimization are always adsorbed parallel to the wall during the imbibition process. However, the desorption of light and medium components on the pore wall occurs as they are transported forward.

However, the phenomenon of a single molecule is not enough to reflect the overall motion characteristics of the adsorption layer. Therefore, the mean-squared displacement (*MSD*) in the X and Z directions of different hydrocarbon components in the first adsorption layer after structural optimization during spontaneous imbibition, as shown in Figure 5, was calculated [38].

(4) $$MSD\left(t\right)={\langle |r_{i}\left(t\right)-r_{i}\left(0\right)|}^{2}\rangle$$

where *r_i_*(*t*) is the position of molecule *i* at time *t*, *r_i_*(0) is the initial position of molecule *i*, and the signal 〈…〉 represents an ensemble average.

For the hydrophilic system, the *MSD* in the X direction of the hydrocarbons is much larger than that in the Z direction (Figure 5a,c), indicating that the first adsorbed layer of shale oil prefers to slip on the wall surface compared to desorption. As the hydrophilicity of the pore wall in SI II decreases, the *MSD* in the X direction of the first adsorption layer of shale oil decreases and is similar to the *MSD* in the Z direction (Figure 5b,d), which means that the slip effect along the wall surface weakens and the adsorbed oil molecules are neither easily desorbed nor diffused on the wall surface. Compared to the strongly hydrophilic system, in the weakly hydrophilic system, more adsorption sites are occupied by medium and heavy components (Figure 3b,e), leading to a slightly enhanced diffusion of light components, but there is no significant change in the *MSD* in the Z direction of the medium and heavy components (Figure 5c,d). Both in the strongly and weakly hydrophilic systems, the heavier hydrocarbon component has a smaller *MSD* in the X and Z direction, which suggests that the heavier component is more inclined to slip along the wall, but its slip effect is diminished. Therefore, the reason why the slip effect on the upper wall of the SI I is more significant is because the adsorption effect of C_20_ on the lower wall is more significant than on the upper wall (Figure 3b). The slip and diffusion of adsorbed oil on the wall surface are not conducive to imbibition and oil displacement. Therefore, how to effectively strip the adsorbed oil (mainly heavy components) during imbibition is a key issue to be considered to improve oil recovery.

### 2.2. Microscopic Advancement Mechanism of Imbibition Front

The driving force for the spontaneous advancement of the meniscus at the molecular level arises from non-bonded interactions between the wall and water molecules, which encompasses both van der Waals and Coulomb interactions. The more hydrophilic walls have higher water–wall interactions and more hydrogen bonds are formed between water molecules and the pore wall (Figure S2a,b). However, the approximately consistent change characteristics of Figure S2a,b indicate that the Coulomb interaction predominantly governs this process due to the highly polar hydrophilic hydroxyl groups present on the pore wall. These hydroxyl groups can form hydrogen bonds with polar water molecules, resulting in a strong Coulombic interaction between water molecules and the pore wall [39]. Furthermore, as shown in Figure 2a,b, the oil phase has a larger *L_S_* in more hydrophilic pores; however, the slip of shale oil on the wall surface is significantly reduced in the weakly hydrophilic system. Therefore, the wettability limitation of the *L_S_* indicates that the advancement of the imbibition front is closely related to the fluid–solid interface behavior.

Previous studies have provided little insight into the microscopic advancement of the imbibition front during the spontaneous imbibition process. Therefore, based on the relevant research on water imbibition into a capillary [40] and visualization results from our MD simulation, we have constructed a schematic diagram depicting the advancement process of the imbibition front during spontaneous imbibition, as shown in Figure 6c. The water in the pore is divided into three parts: “adsorbed water molecules” near the pore wall, “bulk water molecules” in the center of the pore, and “interface water molecules” at the oil–water interface. Shale oil can be divided into “adsorbed oil molecules” near the pore wall and the “bulk phase oil molecules” in the center of the pore.

As shown in Figure 6c, the “bulk water molecules” are almost not affected by the pore wall, but the “adsorbed water molecules” are firmly adsorbed on the pore wall due to the strong Coulomb interaction. Moreover, due to the strong Coulomb interaction of the solid pore wall, the “interfacial water molecules” move forward while approaching the pore wall, eventually becoming “adsorbed water molecules”. Therefore, the “interfacial water molecules” are continuously spread on the pore wall to realize the continuous advancement of the imbibition front and finally the displacement of shale oil out of the pore. To verify the imbibition front advancement process in Figure 6c, two “interfacial water molecules” (M_iw1_ and M_iw2_) and one adsorbed oil molecule (C_20_H_42_) (M_abo_) in SI I at the initial moment were marked (Figure 6a), and then their movement trajectory from 0 to 1000 ps was observed. The visualization trajectory shows that M_iw1_ and M_iw2_ were adsorbed on the pore wall after advancing a certain distance in the pore, eventually becoming immobile “adsorbed water molecules”. Secondly, the observation of the trajectory of M_abo_ reveals that it moves approximately horizontally to the right along the pore wall, which is consistent with the trajectory of C_20_H_42_ in Figure 4.

To quantify the migration process of the three labeled molecules in Figure 6a, the X and Z coordinate values of M_iw1_, M_iw2_, and M_abo_ at different moments were counted, as shown in Figure 6d,e. For M_iw1_, although its X and Z coordinate values fluctuate locally, its X coordinate in general appears first gradually to increase and then stabilize, and its Z coordinate generally appears to gradually decrease and then stabilize, indicating that M_iw1_ flowed along the imbibition direction (X direction) while approaching and adsorbed on the pore wall. The trajectory of M_iw2_ is similar to that of M_iw1_, but the X and Z coordinate values are stabilized more quickly, suggesting that it adsorbs to the pore wall more quickly compared to M_iw1_. The Z coordinate value of M_abo_ fluctuates near the pore wall, and the X coordinate value continues to increase overall, which proves that M_abo_ is continuously displaced along the pore wall to the outside of the pore. The coordinate values of M_iw1_, M_iw2_, and M_abo_ are consistent with the trajectory in Figure 6a. Thus, the microscopic advancement mechanism of the imbibition front during spontaneous imbibition is the competitive adsorption between “interfacial water molecules” and “adsorbed oil molecules”. To determine the reason for the slower diffusion of shale oil at the pore wall in the weakly hydrophilic system, an “interfacial water molecule” in the weakly hydrophilic system was labeled in Figure 6b, which has the same initial position and the same adsorption site on the wall as M_iw2_ during the imbibition process. A comparison of Figure 6a,b reveals that it takes longer for “interfacial water molecules” in a weakly hydrophilic system to adsorb to the same position as M_iw2_, which is the reason why adsorbed oils in the weakly hydrophilic system do not have a significant ability to diffuse on the wall (The positional coordinates over time of “interfacial water molecule” in the X and Z directions in weakly hydrophilic systems also illustrate this issue, as shown in Figure 6f,g).

Because of the asynchronous advancement of the imbibition front, the spontaneous imbibition process in the nanopore can be divided into two stages based on changes in displacement efficiency (*DE*) [41]:

(5) $$DE=\frac{N_{O}-N_{t}}{N_{O}}$$

where *N_O_* represents the number of oil molecules originally in the pore; *N_t_* represents the number of oil molecules in the pore at time *t*.

As shown in Figure 7d, we observe that the *DE* increases approximately linearly in the first stage I (At 0–1621 ps). However, the increase in *DE* in stages II (1621–2519 ps) slows down because the imbibition front reaches the end on the upper walls at 1621ps so that the competitive adsorption of “interfacial water molecules” of the imbibition front and “adsorbed oil molecules” on the pore wall only occurs on the lower wall (inset in Figure 7d). The *DE* in stage I amounts to 69.23%, with a *DE* per unit time of 0.043% ps^−1^. Moreover, the *DE* in stage II amounts to 30.77%, with a *DE* per unit time of 0.034% ps^−1^. That is to say, the displacement velocity in the second stage dropped to 0.77 times that in the first stage. The change in *DE* also proves that spontaneous imbibition is the result of the competitive adsorption between “interfacial water molecules” and “adsorbed oil molecules”.

In addition, the velocity distribution of water molecules in the X direction in both SI I and SI II at 500 ps in Figure 7a,b show significant fluctuations at the oil–water interface. However, the velocity fluctuations of water molecules at the remaining pore positions, except for the oil–water interface region in the X direction, are relatively small. Therefore, the transport of bulk-phase water molecules in the pore is less affected by the wall surface and can be approximated as horizontal advancement. In other words, the essence of spontaneous imbibition oil displacement is also that water molecules outside the pore continuously adsorb and accumulate on the pore wall surface (Figure 7c).

Therefore, the essence of spontaneous oil displacement is the adsorption and accumulation of water molecules outside the matrix pores around the hydroxyl groups on the pore wall, which can be described by the radial distribution function g(*r*). Figure 8a,b depicts the g(*r*) between water molecules and hydroxyl groups on the pore wall. The first peaks of the water molecules in both SI I and SI II were about 0.275 nm away from the hydroxyl groups on the wall, which was close to the distance of the hydrogen bonds [42]. It proves that there are strong hydrogen bonding interactions between the water molecules and the hydroxyl groups on the wall, and the water molecules gather and adsorb around hydroxyl groups because of the hydrogen bond interactions. The peak of g(*r*) and the area under the curve increase with time, suggesting a continuous rise in the coordination number of water molecules surrounding the hydroxyl groups on the pore walls, i.e., water molecules spread forward along the pore walls. By comparing the g(*r*) values in Figure 8a,b, it can be found that the adsorption and aggregation of water molecules on the wall in the weakly hydrophilic system are weakened because of the decrease in the density of hydroxyl groups on the wall. The g(*r*) between oil and water, displayed in Figure 8c,d, is found to be small and remains stable over time, indicating the oil–water interface is stable and there is no significant miscibility between them. Thus, the displacement of oil occurs in a piston-like way. A clear oil–water interface plays a crucial role in preserving optimal interfacial tension, thereby ensuring an effective capillary pressure that serves as the driving force for spontaneous imbibition.

### 2.3. Forced Imbibition under Pressure Difference

Spontaneous imbibition generally occurs under forced pressure (the difference between shut-in pressure and original pore pressure) during the shut-in period [43]. To simulate the forced imbibition process when the pressure difference (Δ*P*, unlike Δ*P* in Equation (3), represents only the difference between shut-in pressure and original matrix pore pressure, the same as below) is 5, 10, and 20 MPa, we apply a matrix pore pressure of 40 MPa to the He plate 2, and different shut-in pressures of 45, 50, and 60 MPa to the He plate 1. The two forced imbibition systems corresponding to systems I and II are named forced imbibition system I (FI I) and forced imbibition system II (FI II), respectively.

The influence of shut-in pressure on imbibition during the shut-in period is vividly described through a series of snapshots at different moments (0, 250, 500, 750, 1000 ps) under varying pressure differences, as illustrated in Figure 9a,b. These snapshots are compared with the corresponding spontaneous imbibition simulation snapshots. It can be intuitively observed that, as the shut-in pressure increases, the imbibition effect also increases accordingly. Notably, the 2-D density distribution shows that the adsorbed oil still present in the pores remains consistently better-adsorbed structures during forced imbibition under pressure difference (Figure 9c).

To quantitatively characterize the stability of the adsorption layer during forced imbibition under pressure difference, the *MSD* in the Z direction of different hydrocarbon components in the first adsorption layer after structural optimization during forced imbibition was calculated, as shown in Figure 10. In forced imbibition under pressure difference, the diffusion performance of the light component change is relatively large, and the diffusion ability of the medium component in the Z direction changes relatively little (Figure 10a,b,d,e). The *MSD* of the heavy components in the Z direction under the effect of pressure difference is not significant (Figure 10c,f), which means that the pressure difference has no significant desorption effect on the heavy components. Therefore, the axial pressure difference leads to irregular changes in adsorption diffusion properties but does not effectively strip the adsorbed layer of shale oil (especially the heavy component).

Further observation of the imbibition front of the simulated snapshots of forced imbibition in Figure 9a,b shows that with the increase in the pressure difference, the degree of curvature of the meniscus at the imbibition front tends to weaken (i.e., the value of *θ_d_* increases with the increase in shut-in pressure), and it is especially obvious at 20 MPa. In the weakly hydrophilic system, the phenomenon of boundary water lagging behind the water in the bulk phase was observed even under the pressure difference of 20 MPa, which means that the morphology of the meniscus at the imbibition front changes from concave to convex on the aqueous side. This may be due to wetting hysteresis caused by a pressure difference [44].

**Figure 11:**
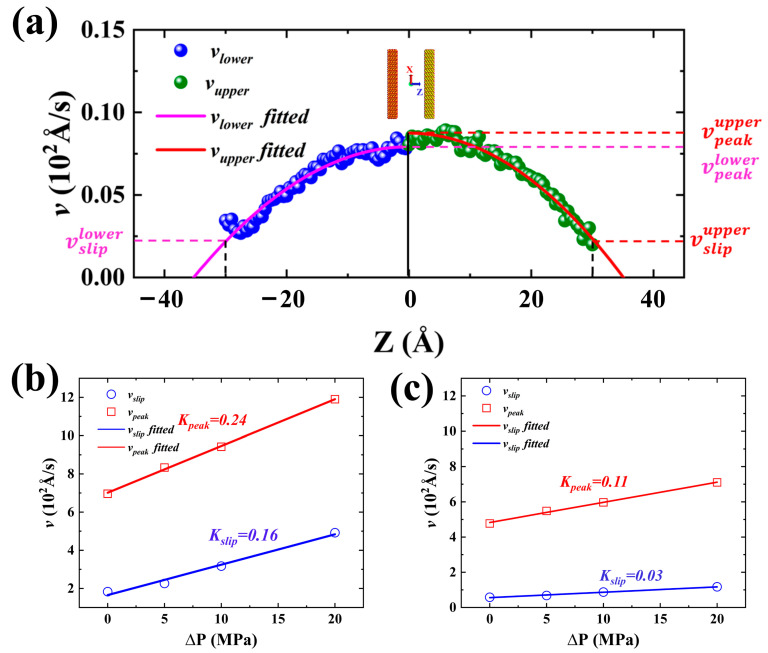
(**a**) Velocity distribution of shale oil and schematic of slip velocity ($v_{slip}^{lower}$ and $v_{slip}^{upper}$) and peak velocity ($v_{peak}^{lower}$ and $v_{peak}^{upper}$) obtained by fitting velocity distribution of shale oil. (**b**) $v_{slip}$ and $v_{peak}$ of shale oil at different pressure differences, and slope obtained by linearly fitting $v_{slip}$ and $v_{peak}$ of shale oil in FI I. (**c**) $v_{slip}$ and $v_{peak}$ of shale oil at different pressure differences, and slope obtained by linearly fitting $v_{slip}$ and $v_{peak}$ of shale oil in FI II.

**Figure 1:**
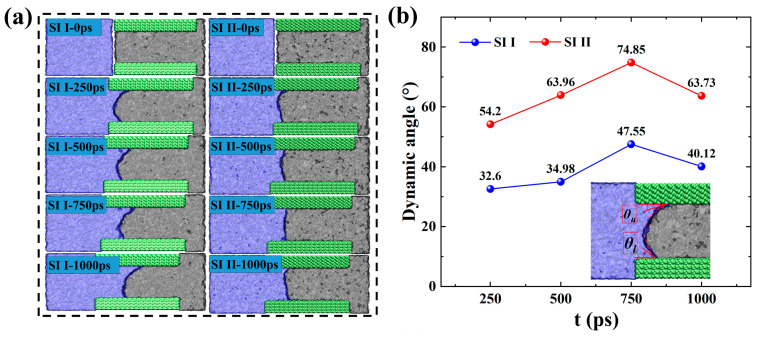
(**a**) Simulation snapshots of SI I and SI II, and (**b**) corresponding dynamic contact angles at several different moments. In the snapshots of the imbibition systems in (**a**), the blue zone represents water molecules, the green zone represents the SiO_2_ wall surface, and the gray zone represents hydrocarbon molecules, respectively, the same as below. The inset in (**b**) shows a schematic diagram of the *θ_u_* on the upper pore wall and *θ_l_* on the lower pore wall.

**Figure 2:**
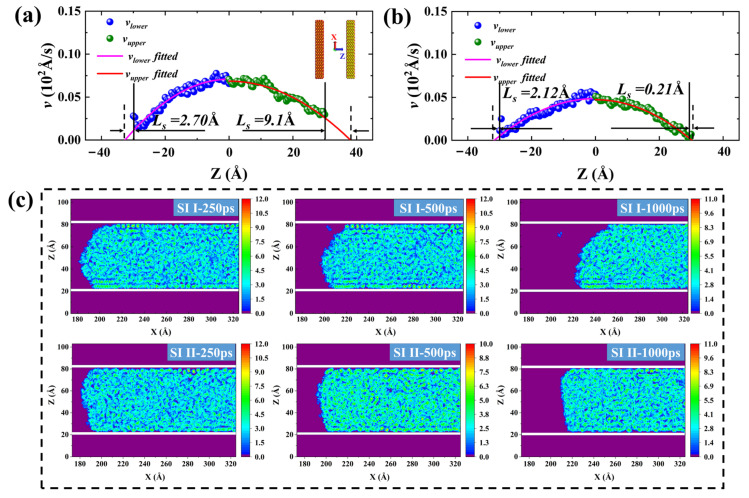
Velocity distribution of shale oil in the Z direction and *L_S_* obtained by fitting velocity distribution in the (**a**) SI I and (**b**) SI II, respectively. (**c**) 2-D density distribution of shale oil at different moments during spontaneous imbibition in SI I and SI II. The purple zone in (**c**) represents the background of the 2-D density distribution of shale oil, the long white bar represents the quartz wall surface, and the rest of the zone is the 2-D density distribution of shale oil, the same as below.

**Figure 3:**
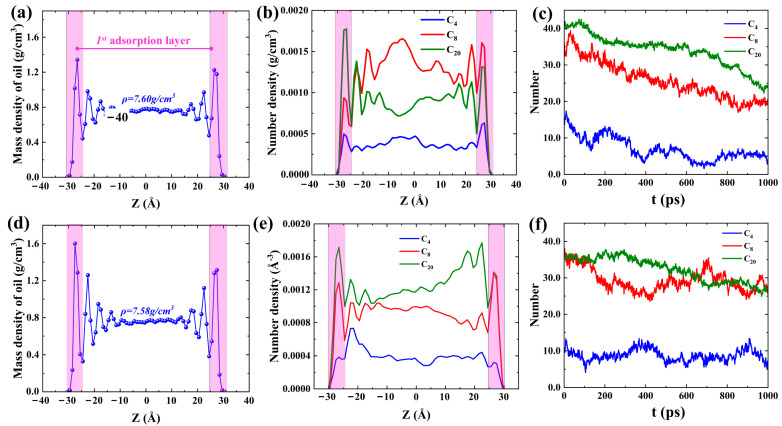
The mass density distribution of shale oil in the pores of (**a**) SI I and (**d**) SI II after 3 ns of structural optimization, respectively. The number density distribution of different hydrocarbon components in the pores of (**b**) SI I and (**e**) SI II after 3 ns of structural optimization, respectively. The number of changes of different hydrocarbon components in the first adsorption layer with time during spontaneous imbibition in (**c**) SI I and (**f**) SI II, respectively. In (**a**,**b**,**d**,**e**), the long purplish red bar represents the first adsorption layer.

**Figure 4:**
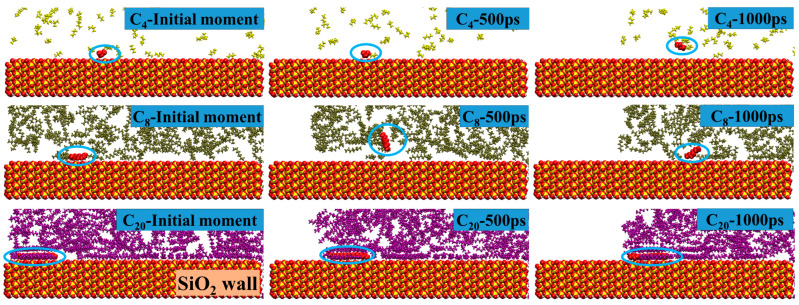
Migration trajectories of C_4_, C_8_, and C_20_ in the first adsorption layer after structural optimization during spontaneous imbibition. The red molecules in the blue circle are labeled as different hydrocarbon molecules in the first adsorption layer after structural optimization, and the yellow, tan, and purple molecules represent C_4_, C_8_, and C_20_, respectively, the same as below.

**Figure 5:**
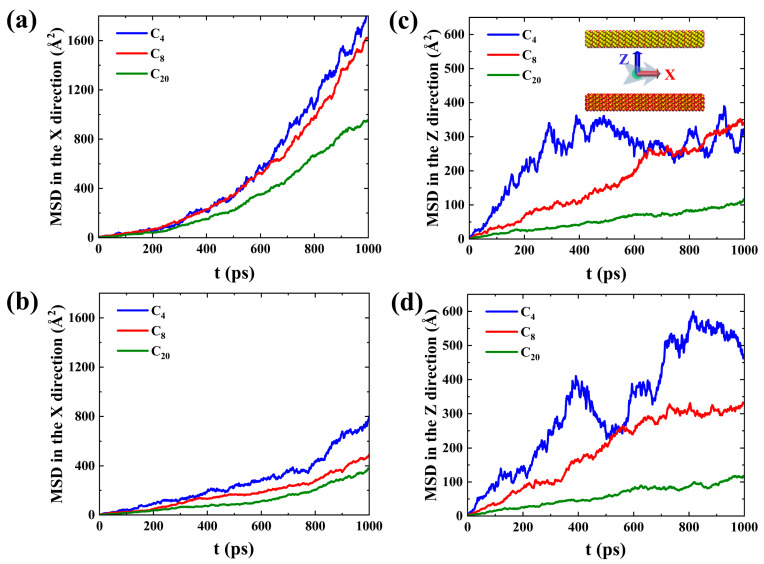
*MSD* in (**a**) X and (**b**) Z directions of different hydrocarbon components in the first adsorption layer after structural optimization in SI I during spontaneous imbibition, respectively. *MSD* in (**c**) X and (**d**) Z directions of different hydrocarbon components after structural optimization in the first adsorption layer in SI II during spontaneous imbibition, respectively.

**Figure 6:**
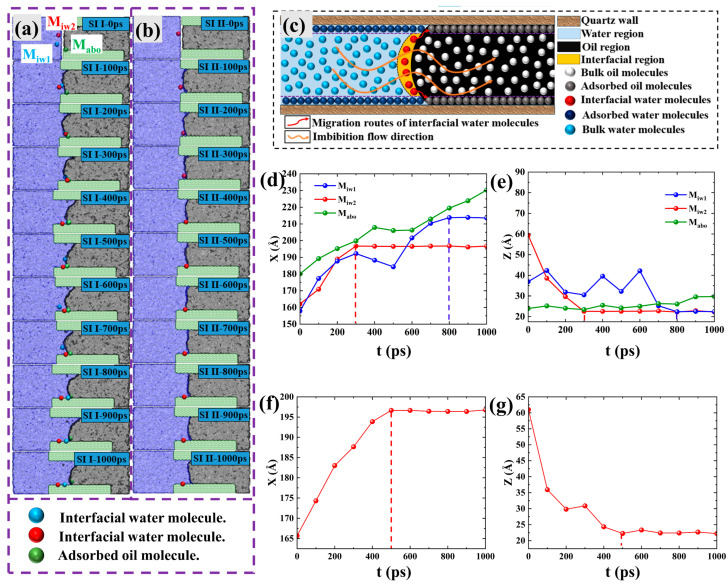
(**a**) Migration trajectories of “interfacial water molecules” and “adsorbed oil molecules” in SI I. (**b**) Migration trajectories of “interfacial water molecules” in SI II; (**c**) Schematic of the advancing imbibition front. Position coordinates of two “interfacial water molecules” (M_iw1_ and M_iw2_) and one “adsorbed oil molecule” (M_abo_) in the (**d**) X and (**e**) Z directions in SI I at several different moments, respectively. Position coordinates of one “interfacial water molecule” in the (**f**) X and (**g**) Z directions in SI II at several different moments, respectively.

**Figure 7:**
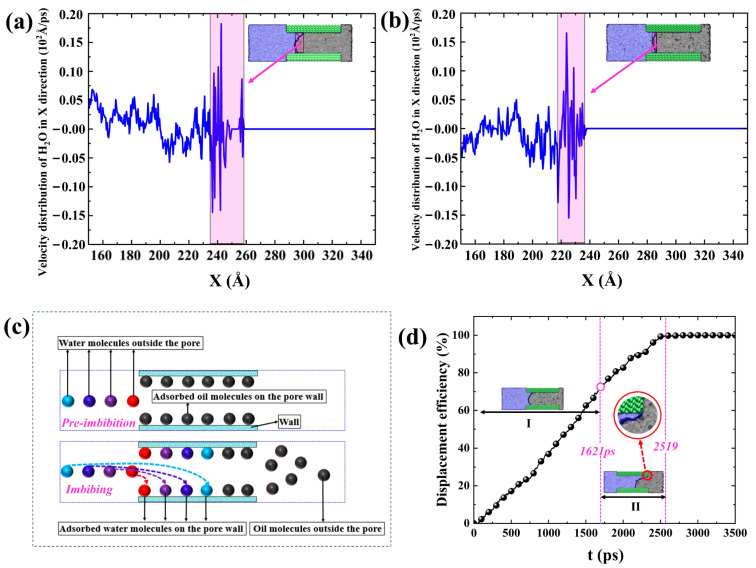
Velocity distribution of water molecules in the X direction in (**a**) SI I and (**b**) SI II at 500 ps, respectively. (**c**) Schematic diagram of the migration trajectories of water molecules outside the pore. (**d**) Imbibition *DE* of SI I. The purplish red bar represents the oil–water interface region in (**a**,**b**). Red, purple, dark blue, and light blue balls in (**c**) represent water molecules outside the pore successively adsorbed to the pore wall, respectively, and the black balls represent hydrocarbon molecules. The inset in the red circle in (**d**) means the imbibition front reaches the right end of the nanopore on the upper walls.

**Figure 8:**
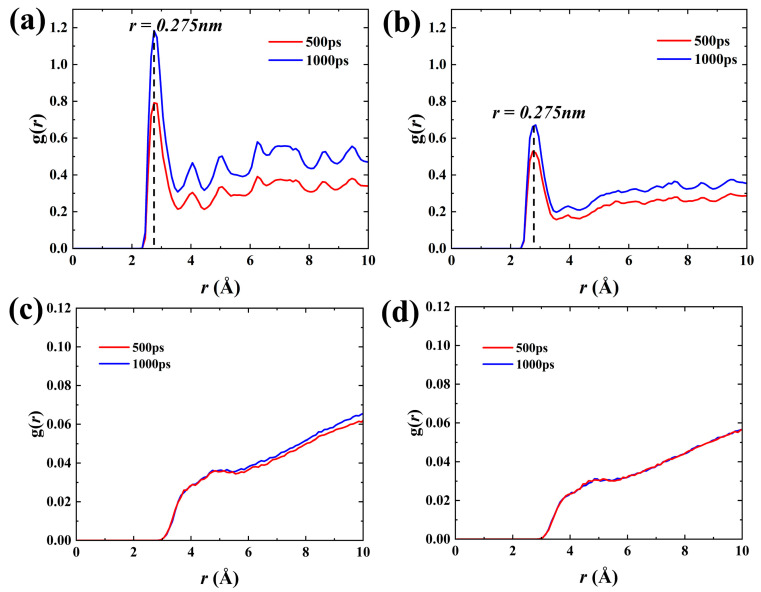
Radial distribution function g(*r*) of O (hydroxyl) and O (H_2_O) atoms in the (**a**) SI I and (**b**) SI II, respectively. g(*r*) of O (H_2_O) and C (hydrocarbons) atoms in the (**c**) SI I and (**d**) SI II.

**Figure 9:**
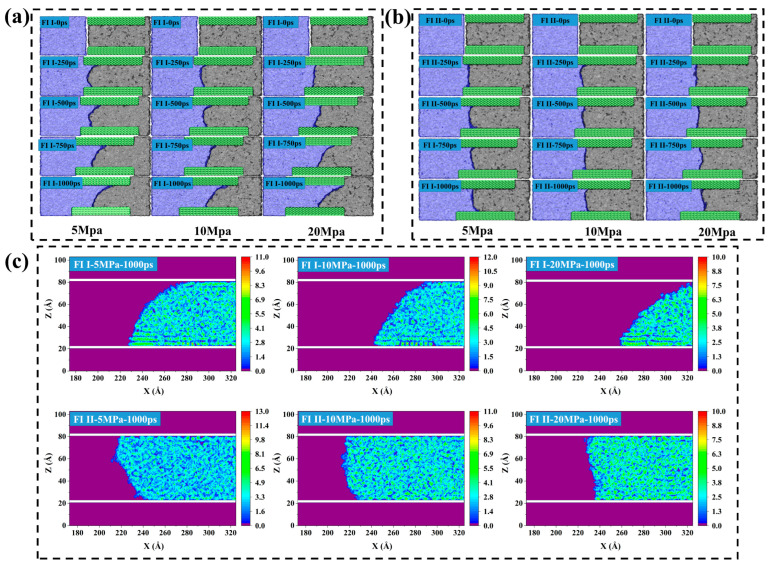
Snapshots of (**a**) FI I and (**b**) FI II under different pressure differences at several moments. (**c**) 2-D density distribution of shale oil at 1000 ps in FI I and FI II. The purple zone in (**c**) represents the background of the 2-D density distribution of shale oil, the long white bar represents the quartz wall surface, and the rest of the zone is the 2-D density distribution of shale oil.

**Figure 10:**
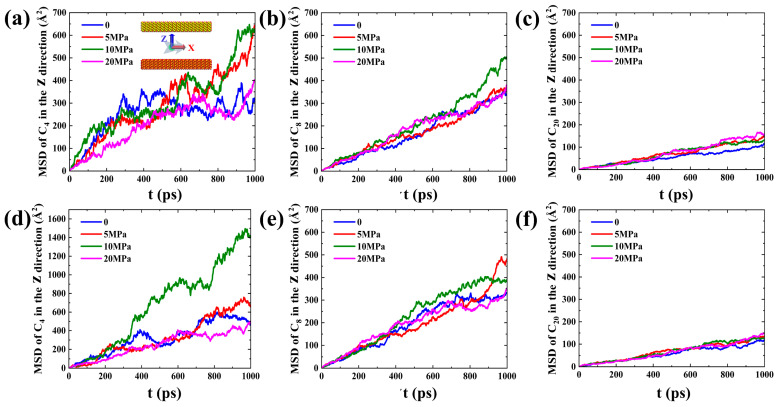
*MSD* in the Z direction for (**a**) C_4_, (**b**) C_8_, and (**c**) C_20_ in the first adsorption layer after structural optimization in FI I during forced imbibition, respectively. *MSD* in the Z direction for (**d**) C_4_, (**e**) C_8_, and (**f**) C_20_ in the first adsorption layer after structural optimization in FI II during forced imbibition, respectively.

Because the uneven distribution of mixed crude oil results in an asynchronous advancement of the imbibition front and a difficulty in accurately measuring the dynamic contact angle, other methods are needed to quantitatively characterize the wetting hysteresis phenomenon. The wetting hysteresis phenomenon is closely related to the velocity distribution of the fluid of the nanopore in the Z direction. Thus, the slip velocity and peak velocity variations can quantitatively characterize the wetting hysteresis phenomenon and can be obtained by fitting the velocity distribution of shale oil using Equation (3), as shown in Figure 11a. Considering the asynchronous advancement of the imbibition front and the accuracy of the results, both the slip velocity ($v_{slip}$) and peak velocity ($v_{peak}$) are obtained by arithmetic averaging, as shown in Equations (6) and (7).

(6) $$v_{slip}=\frac{v_{slip}^{lower}+v_{slip}^{upper}}{2}$$

(7) $$v_{peak}=\frac{v_{peak}^{lower}+v_{peak}^{upper}}{2}$$

where $v_{slip}^{lower}$, $v_{slip}^{upper}$, and $v_{slip}$ are the slip velocities of the shale oil on the lower and upper walls, and their arithmetic mean, respectively; $v_{peak}^{lower}$, $v_{peak}^{upper}$, and $v_{peak}$ are the peak velocities of shale oil on the lower and upper sides of the center of the pore, and their arithmetic mean, respectively. The fitting results for systems I and II are presented in Figure 11b and Figure 11c, respectively.

Linear fitting again of $v_{slip}$ and $v_{peak}$ obtained by Equations (6) and (7) can obtain the slopes *K_peak_* and *K_slip_*, respectively representing the change rate of peak velocity and slip velocity with pressure difference. Both $v_{slip}$ and $v_{peak}$ increase faster for the strongly hydrophilic system than for the weakly hydrophilic system, representing a more significant promotion of the imbibition in the strongly hydrophilic system by the pressure difference. In both strongly and weakly hydrophilic systems, *K_peak_* is greater than *K_slip_*, leading to an increase in the dynamic contact angle (i.e., wetting hysteresis). Further comparison of the ratios of *K_peak_* and *K_slip_* (i.e., $\frac{K_{peak}}{K_{slip}}$) for the different imbibition systems, respectively, show that the ratio $\frac{K_{peak}}{K_{slip}}$ of the weakly hydrophilic system is greater than that of the strongly hydrophilic system. This finding suggests that the difference between the $v_{peak}$ and $v_{slip}$ in the strongly hydrophilic system changes less than that of the weak hydrophilic system, which means that the wetting hysteresis phenomenon of the weak hydrophilic system is more significant.

To further clarify the wettability and pressure difference correlation because of the wetting hysteresis, we have carried out certain theoretical derivations and analyses. Based on the reference [45], when forced imbibition occurs after shut-in, the equilibrium of forces on the fluid in the confined capillary can be expressed as:

(8) $$F_{c}+F_{P}+f+F_{i}+F_{g}=0$$

where *F_c_* is the capillary force; *F_P_* is the displacement force; *f* is the viscous force generated by the friction between fluid molecules and between fluid molecules and the solid pore wall; *F_i_* is the inertial force; and *F_g_* is gravity. Because the matrix pore width of tight shale reservoirs is extremely small and much smaller than the imbibition height, the influence of *F_g_* can be ignored [46]. Secondly, for pores with extremely small radii, *f* dominates and the *F_i_* can be ignored [47]. Then Equation (8) can be simplified to the following:

(9) $$F_{c}+F_{P}+f=0$$

Assuming that the width between two walls, length in the Y direction, and length in the X direction of the slit-pore are *w*, *l*, and *L*, respectively, it is possible to convert displacement force *F_P_* into *wl*Δ*P*. According to the Young–Laplace Equation [48], the *F_c_* in a single slit-pore is the following:

(10) $$F_{c}=\frac{2\sigma cos\;\theta }{w}wl=2\sigma lcos\theta$$

where *θ* is the contact angle of the oil–water–wall three-phase system, which is assumed to be equal to the test results of static contact angle and is not affected by other factors; *σ* is the oil–water interfacial tension. The total *f* of oil and water in a single pore is:

(11) $$f=2xl\tau_{w}+2l\left(L-x\right)\tau_{o\;}$$

where *x* is the position of the oil–water interface in the X direction; *τ_w_* and *τ_o_* are the shear stress of water and oil, respectively, which can be expressed as the following [49]:

(12) $$\begin{array}{c} \tau_{w}=-\frac{12\mu_{w}}{w}v\\ \tau_{o}=-\frac{12\mu_{o}}{w}v \end{array}$$

where v is the imbibition velocity, and *μ_w_* and *μ_o_* are the viscosity of water and oil, respectively. After bringing Equations (10)–(12) into Equation (9) and rewriting v as the derivative *dx*/*dt* of *x* with respect to time *t*, Equation (9) is transformed into the following:

(13) $$\Delta Pw^{2}+2\sigma wcos\theta -24\left[x\mu_{w}+\left(L-x\right)\mu_{o}\right]\frac{dx}{dt}=0$$

After integrating Equation (13) under the initial condition of *x* = 0 and *t* = 0 (*dt* are the integration variable), we get the following:

(14) $$12\left(\mu_{w}-\mu_{o}\right)x^{2}+24\mu_{o}Lx-\left(\Delta Pw^{2}+2\sigma wcos\;\theta \right)t=0$$

Additionally, it should be noted that the shale oil model used in this study does not account for components such as C_20+_ and asphaltenes, implying that its viscosity could be lower under reservoir conditions. As previously mentioned, the oil *DE* primarily exhibits a linear change during the imbibition process before the imbibition front reaches the outlet. Considering the low viscosity of the Jimsar shale oil under reservoir conditions, for simplification, the viscosity of shale oil under reservoir conditions can be approximated as *μ* = *μ_w_* = *μ_o_* = 0.36 mPa·s, i.e., the viscosity of water under the same conditions obtained from the NIST database. Consequently, Equation (14) can be simplified as follows:

(15) $$24\mu Lx-\left(\Delta Pw^{2}+2\sigma wcos\;\theta \right)t=0$$

Then the imbibition velocity at pressure difference is

(16) $$v=\frac{x}{t}=\frac{\left(\Delta Pw^{2}+2\sigma wcos\;\theta \right)}{24\mu L}$$

The pressure differences are equal to 0, 5, 10, and 20 MPa, respectively, in this study. When the pressure difference is equal to 0, Equation (16) is converted to v=σwcos θ/12μL, indicating that the spontaneous imbibition velocity is constant during spontaneous imbibition under reservoir conditions. This may be the reason why the oil DE changes approximately linearly under reservoir conditions before the imbibition front reaches the outlet. Some parameters of Equation (16) are: The *σ* under reservoir conditions obtained by MD simulation is 55.44 mN/m (Figure S3) (The results indicate a good match between the simulation and experimental value of 53.2 mN/m [50]); The static contact angles (*θ_S_*) for the strongly and weakly hydrophilic walls are 53.9° and 78.97°, respectively (Figure S5d); The *L* and *w* are 15 and 6 nm (see the Section 3 and Figure S4).

The imbibition velocity when the pressure difference is equal to 0 is called the spontaneous imbibition velocity ($v_{SI}$), and the imbibition velocity when the pressure difference is equal to 5, 10, and 20 MPa is called the forced imbibition velocity ($v_{FI}$). Assuming that, when capillary imbibition occurs, the *θ* does not change with other factors such as the pressure difference, the ratio (*R*) of forced imbibition to spontaneous imbibition velocity at the pressure difference is as follows:

(17) $$R=\frac{v_{FI}}{v_{SI}}=\frac{\Delta Pw^{2}}{2\sigma wcos\;\theta_{s}}+1$$

From Equation (17), as assumed earlier in the subsection, the three-phase contact angle *θ* is not affected by the pressure difference and is always equal to the *θ_S_*. Therefore, the *R* of the weakly hydrophilic system should be greater than that of the strongly hydrophilic system at the same pressure differences. However, the calculated *R* as shown in Table 1 shows that the *R* of the strongly hydrophilic system is larger than that of the weakly hydrophilic system, which further proves that the pressure difference leads to the occurrence of the wetting hysteresis phenomenon, i.e., *θ* changes under the influence of the pressure difference.

The imbibition velocity at different pressure differences obtained by substituting the value of these parameters mentioned above into Equation (16) is renamed as the theoretical imbibition velocity ($v_{th}$) under different pressure differences. Meanwhile, we rename the imbibition velocity obtained through the MD simulation in Table 1 as the MD simulation velocity ($v_{MD}$). The calculation method of the imbibition velocity obtained through MD simulation involves taking the average value of the fitted velocity curve in Figure 11a. Although the theoretically calculated and MD-simulated values do not necessarily correspond to the real imbition velocity, their change characteristics can quantitatively reflect a certain imbibition mechanism.

**Figure 12:**
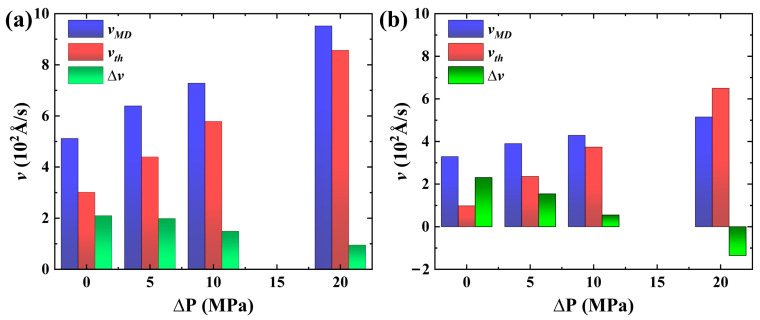
$v_{MD}$, $v_{th}$, and Δv in (**a**) strongly hydrophilic and (**b**) weakly hydrophilic systems, respectively.

Figure 12a,b illustrates the values of $v_{MD}$ and $v_{th}$, as well as the difference (Δv) (Δv =$v_{MD}$ − $v_{th}$) between them at different pressure differences. The results show basically that $v_{MD}$ and $v_{th}$ exhibit the same order of magnitude, confirming the reliability of the simulation outcomes. In imbibition systems I and II, as the pressure difference increases, both $v_{MD}$ and $v_{th}$ gradually increase, while Δv continues to decrease. The results demonstrate that an increase in pressure difference can enhance the imbibition effect, while also leading to a reduction in the effective imbibition velocity relative to the theoretical value because of the wetting hysteresis phenomenon. Moreover, Δv decreases more rapidly in the weakly hydrophilic system relative to the strongly hydrophilic system, suggesting that a more pronounced wetting hysteresis phenomenon leads to a more pronounced loss of the imbibition velocity relative to theoretical values. The loss of imbibition velocity relative to the theoretical velocity is caused by the reduction in capillary pressure due to wetting hysteresis. Consequently, excessively high imbibition pressure impedes the maximization of economic benefits and reduces the underutilization of natural power.

## 3. Models and Methodology

### 3.1. Model System

All molecular models were constructed through Materials Studio software (version: Materials Studio 2020) developed by Accelrys Company in the San Diego, CA, USA. The molecular model constructed in this article mainly includes solid quartz walls, nanopores composed of quartz walls, and fluid components (shale oil and water).

China’s nonmarine shale is typically composed of variable amounts of detrital minerals such as quartz, feldspar, and calcite with content generally higher than 40%, and clay minerals such as smectite, illite, and kaolinite [51]. In addition, the brittle mineral content affects the microcrack development, oil-bearing capacity, and fracturing stimulation techniques. Higher content of fragile minerals such as quartz and feldspar, and lower content of clay minerals such as kaolinite and smectite, enhance rock brittleness. High brittleness means that intrinsic fractures and induced fractures are prone to appear in the shale under the action of external forces, which would facilitate shale oil recovery [52,53]. Therefore, quartz is one of the key matrix minerals in the shale oil extraction process.

The research object of this study is the shale oil reservoirs of the Lucaogou Formation in the Jimsar Sag, Xinjiang oilfield. The reservoir has the geological characteristics of integrated source and storage, and a dispersed “sweet spot”, with an average reservoir temperature of 80 °C [54,55] and an original formation pressure of about 40 MPa [56,57]. The Luchaogou Formation (P_2_l) consists of upper (P_2_l_2_) and lower (P_2_l_1_) sections, each with its own “sweet spot” enrichment sections. Scholars [58,59] have conducted mineral components analysis on shale core samples from the Jimsar reservoir by using a device of X-ray diffraction. The results show that the mineral composition of the Lucaogou Formation shale matrix is complex and diverse, but quartz has the highest relative content and plays an important role. Quartz is often used to simulate inorganic nanopores and is generally hydrophilic [41,60,61,62]. Furthermore, it is mentioned in *physics of petroleum reservoirs* that spontaneous imbibition can only occur in hydrophilic capillaries. Therefore, considering that minerals themselves were not one of the research variables, in summary, a single mineral quartz is used in this work to represent shale reservoir matrix pores. The initial quartz unit cell SiO_2_ Quartz was taken from the structure database of Materials Studio software and then was cleaved along the ($0\bar{10}$) crystal face. After being cleaved, the crystal face was replicated to get the final quartz wall. The complexity and randomness of the mineral distribution in shale reservoirs lead to a heterogeneous distribution of wettability as well [63]. To characterize the different hydrophilicity of shale reservoirs, silica was modified concerning existing research methods. The strongly hydrophilic modification of the wall surface (named W_A_) was first realized by adding hydrophilic group hydroxyl (-OH) to all chemically unsaturated Si atoms of the $0\bar{10}$ crystal surface (surface in contact with oil), with a surface hydroxyl density of 9.6 nm^−2^, which was consistent with the results of the crystal chemistry calculations (5.9–18.8 nm^−2^) [64]. Then, hydroxyl hydrogens of the $0\bar{10}$ crystal surface were substituted with a certain amount of hydrophobic group methyl (-CH_3_) to obtain the weakly hydrophilic wall surface (named W_B_). To make the wall surface as homogeneous as possible, all the hydroxyl and methyl groups were evenly distributed on the wall surface of the quartz. The hydroxyl and methyl density on the surface in contact with the oil of the modified quartz wall was 7.2 nm^−2^ and 2.4 nm^−2^. The back surface and left and right end surfaces of the quartz walls were all fully hydroxylated. Considering that spontaneous imbibition in oil-wet (i.e., hydrophobic) pores cannot occur effectively, only hydrophilic matrix pores of the shale reservoir are considered in this paper. Each wall has a thickness of 21.6 Å and dimensions in the X and Y directions of 150 Å and 29.5 Å, respectively. Quartz walls with different hydrophilicity are presented in Figure S4.

**Figure 13:**
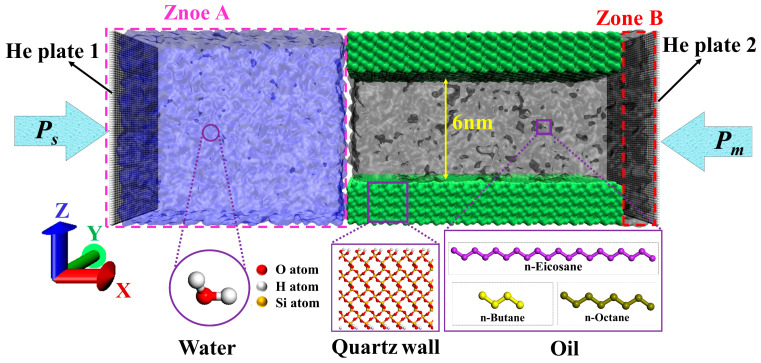
Snapshot of the initial configuration of the imbibition model.

Before conducting MD imbibition simulations, the static wettability of different walls was first quantitatively characterized (The corresponding results are presented in Figure S5). Then, on this basis, the molecular model of imbibition oil displacement was constructed, as shown in Figure 13. The nanopore representing a slit-shaped quartz nanopore in the shale matrix consisted of two quartz walls with the same modification, as shown in Figure S4. Zones A and B represent microfracture or macropore to which the pore is connected at the water-injection end and microfracture or macropore to which the pore is connected at the non-water-injection end, respectively. Furthermore, previous studies have shown that the Jimsar shale reservoir develops abundant nanopores [65,66,67]. The Young–Laplace equation [48] shows capillaries with smaller radii have more significant imbibition effects. Secondly, MD simulations are subject to limited computing resources. Therefore, comprehensively considering the computational resources and the significant imbibition effect, the vertical distance (*w*) between their wall surfaces in the Z direction is set to 60 Å, and the two walls of the pore were placed parallel to the X-Y plane. For the shale oil in the pore, considering the complexity of shale oil components, a single oil component cannot truly reflect the occurrence and flow state of shale oil in the pore. According to Table S2, in this study, a ternary system composed of n-Butane (n-C_4_H_10_), n-Octane (n-C_8_H_18_), and n-Eicosane (n-C_20_H_42_) was selected to represent shale oil in the lower “sweet spot” of the Lucaogou Formation in the Jimsar Sag, Xinjiang oilfield. n-Butane (n-C_4_H_10_), n-Octane (n-C_8_H_18_), and n-Eicosane (n-C_20_H_42_) respectively represent the light, medium, and heavy components in Jimsar shale oil. The molar fractions and number of molecules of each hydrocarbon component of the shale oil model in the pore are shown in Table S2. Water molecules representing the injected water in the microfracture or macropore to which the pore is connected, i.e., fluid surrounding the matrix, are placed to the left of the pore. Before the imbibition oil displacement simulation, the shale oil was fully adsorbed on the pore wall through 3 ns equilibrium molecular dynamics (EMD) at reservoir conditions (353.15 K and 40 MPa), thereby obtaining a reasonable shale oil density. During the imbibition simulation, He plates, which are parallel to the Z-Y plane, were placed on the left and right sides of the oil–water–wall system, and the imbibition oil displacement process was controlled by controlling the pressure attached to the He plate. Furthermore, plates 1 and 2 can only be moved parallel to direction X. In this work, it is assumed that the shut-in pressure is equal to the fluid pressure around the matrix. The pressure on He plate 2 always maintains the matrix pore pressure (*P_m_*) of 40 MPa, while the pressure exerted on He plate 1 representing the shut-in pressure (*P_s_*) is greater than or equal to *P_m_*. When the pressure on He plate 1 is equal to *P_m_*, water is spontaneously imbibed into the pore. When the pressure applied to He plate 1 exceeds *P_m_*, it is the process of forced imbibition oil displacement. The simulation time of imbibition oil displacement is 1 ns.

### 3.2. Simulation Details

Different force fields have different characteristics and applicable conditions. Therefore, choosing an appropriate force field for a specific research system is key to the accuracy and reliability of MD simulation results. In this paper, we use different force field potential energy parameters for different molecular groups. The ClayFF force field was developed by Randall T et al. [68] for hydrates and multi-component minerals and their interaction with fluid interfaces and has been successfully applied to hydroxylated silica surface-fluid systems [69,70,71,72]. Thus, this paper uses the ClayFF force field to describe the silica crystal and surface hydroxyl groups. Referring to the research of Aleksandr Abramov, the remaining surface methyl groups are described by the DREIDING force field [73]. The OPLS-AA force field [74] is used to describe n-hydrocarbons in shale oil. The OPLS force field can accurately describe hydrocarbon molecules’ thermodynamic properties and structural characteristics [75]. The SPC/E potential energy model [76] is used to describe water molecules. SPC/E can reproduce the dynamic physical behavior of water molecules on the interface and has good compatibility with the ClayFF force field and OPLS-AA force field [77,78,79,80]. The He plate and quartz wall are considered rigid bodies. Periodic boundary conditions are applied in all directions. This article uses Large-scale Atomic/Molecular Massively Parallel Simulator (LAMMPS) open-source program software (version: 28 Mar 2023) to perform MD simulations [81], with a time step of 1fs. The simulation results were visualized using Visual Molecular Dynamics (VMD) software (version: 1.9.4) [82]. Intermolecular non-bonded interactions (*E_nonbonded_*) can be described by the standard 12-6 Lennard-Jones potential (*F_LJ_*) and Coulomb potential, respectively (*E_coulomb_*) [70,83].

(18) $$E_{nonbonded}=E_{LJ}+E_{coulomb}=4\varepsilon_{ij}\left[{\left(\frac{\sigma_{ij}}{r_{ij}}\right)}^{12}-{\left(\frac{\sigma_{ij}}{r_{ij}}\right)}^{6}\right]+\frac{1}{4\pi \varepsilon_{0}\varepsilon_{r}}\frac{q_{i}q_{j}}{r_{ij}}$$

where *r_ij_* stands for the distance between two atoms *i* and *j*; *ε_ij_* and *σ_ij_* represent the LJ well depth and the zero-potential distance between atoms *i* and *j*, respectively; *ε*_0_ and *ε_r_* represent the dielectric constant of a vacuum and relative permittivity of the medium, respectively. And *q_i_* and *q_j_* represent the charge of atoms *i* and *j*, respectively. The cutoff radius was set to be 10 Å. All MD simulations were performed in the NVT ensemble. The interactions between different atoms are calculated using the Lorentz–Berthelot mixing criterion [84]:

(19) $$\sigma_{ij}=\frac{1}{2}\left(\sigma_{ii}+\sigma_{jj}\right)$$

(20) $$\varepsilon_{ij}=\sqrt{\varepsilon_{ii}\varepsilon_{jj}}$$

## 4. Conclusions

This study uses MD simulation to elucidate the microscopic mechanism of water imbibition and oil displacement in the different hydrophilic pores of the shale matrix under different shut-in pressures. The main conclusions are as follows:

(1) The slip phenomenon suggests that the strong attraction between hydrocarbons and quartz walls leads to the inability of water molecules to effectively peel off the oil phase. The heavy hydrocarbon component in the adsorbed layer tends to slip on the walls and the light component tends to desorb from the pore wall. However, the heavier hydrocarbon components have poorer slip ability on the wall. Weak wall hydrophilicity and slip of shale oil on the wall surface can significantly reduce the efficiency of imbibition displacement. How to effectively strip the adsorbed oil (especially heavy components) during imbibition is a key issue to be considered to improve oil recovery.

(2) The displacement of oil occurs in a piston-like way during spontaneous imbibition. The microscopic advancement mechanism of the imbibition front is the competitive adsorption between “interfacial water molecules” at the imbibition front and “adsorbed oil molecules” on the pore wall. The essence of spontaneous imbibition is the adsorption and aggregation of water molecules onto the hydroxyl groups present on the pore wall. The better hydrophilicity of the matrix pore’s wall facilitates higher levels of adsorption and accumulation of water molecules on the pore wall and requires less time for “interfacial water molecules” to compete with “adsorbed oil molecules” because interfacial water molecules have a stronger competitive adsorption effect on the more hydrophilic pore.

(3) During the forced imbibition process, as the pressure difference increases, the imbibition effect gradually increases. The pressure difference acts mainly on the bulk oil, thus causing wetting hysteresis to occur. Meanwhile, shale oil still existing in the pore always maintains a good, stratified adsorption structure. During forced imbibition, the actual capillary pressure gradually decreases because of the wetting hysteresis phenomenon and there is a loss in the imbibition velocity relative to the theoretical value. Simultaneously, the decline in hydrophilicity further weakens the synergistic effect on imbibition of pressure difference because of the more pronounced wetting hysteresis. Consequently, excessively high shut-in pressure impedes the maximization of economic benefits and reduces the underutilization of natural power.

The study presented in this article investigates the microscopic mechanism and process of imbibition oil displacement in matrix shale pores under different wettabilities and shut-in well pressures. The findings offer valuable insights into improving imbibition oil displacement methods during the shut-in period, optimizing shut-in time and operation after the shale reservoir has been hydraulically fractured, and eventually enhancing production in shale oil reservoirs.

## Figures and Tables

**Table 1:** $v_{MD}$ and *R* under different pressure differences in imbibition systems I and II.

Δ*P* (Mpa)	$v_{MD}$ (10^2^ Å/s)		*R*	
	System I	System II	System I	System II
0	5.12	3.29	1.00	1.00
5	6.39	3.90	1.24	1.19
10	7.28	4.29	1.42	1.30
20	9.52	5.15	1.85	1.56

## Data Availability

The raw/processed data required to reproduce these findings cannot be shared at this time, as the data also forms part of an ongoing study.

## Supplementary Materials

The following supporting information can be downloaded at: https://www.mdpi.com/article/10.3390/molecules29051112/s1, Figure S1: The interaction energy between oil and wall during structural optimization of the oil-pore systems of imbibition systems I and II; Figure S2: (a) The interaction energy between water molecules and wall, and (b) Hydrogen bond number as a function of time *t* in spontaneous imbibition systems I-II, respectively; Figure S3: Molecular model for calculating the interfacial tension of pure oil and water systems; Figure S4: Quartz wall sizes and different modified wall surfaces with different wettability; Figure S5: (a) Initial and (b) equilibrium configuration for static contact angle testing, respectively. (c) Schematic diagram of static contact angle calculation method. (d) Static wetting angle of W_A_ and W_B_; Figure S6: The mass fraction of hydrocarbons with different numbers of carbon atoms of (a) JHW05815 Oil Sample, (b) JHW07121 Oil Sample, and (c) J41 Oil Sample; Table S1: The average mass fraction of light, heavy, and medium hydrocarbon components of the Jimsar shale oil; Table S2: The mole fractions and number of each component of the shale oil model; Table S3: Atomic charge and detailed interaction parameter of SiO_2_, oil, and water. References [59,63,85,86] are cited in the Supplementary Materials.

## References

[1] Xia D., Yang Z., Gao T., Li H., Lin W. (2021). Characteristics of micro-and nano-pores in shale oil reservoirs. J. Pet. Explor. Prod..

[2] Dai C., Wang K., Liu Y., Fang J., Zhao M. (2014). Study on the reutilization of clear fracturing flowback fluids in surfactant flooding with additives for Enhanced Oil Recovery (EOR). PLoS ONE.

[3] Shaibu R., Guo B. (2021). The dilemma of soaking a hydraulically fractured horizontal shale well prior to flowback—A decade literature review. J. Nat. Gas Sci. Eng..

[4] Hu Y., Ren F., Li J., Wu Z., Peng H., Hou J. (2021). Effect of dynamic imbibition on the development of ultralow permeability reservoir. Geofluids.

[5] Sobhani A., Ghasemi Dehkordi M. (2019). The effect of nanoparticles on spontaneous imbibition of brine into initially oil-wet sandstones. Energy Sources Part A Recovery Util. Environ. Eff..

[6] Morrow N.R., Mason G. (2001). Recovery of oil by spontaneous imbibition. Curr. Opin. Colloid Interface Sci..

[7] Nguyen D., Wang D., Oladapo A., Zhang J., Sickorez J., Butler R., Mueller B. (2014). Evaluation of Surfactants for Oil Recovery Potential in Shale Reservoirs.

[8] Hatiboglu C.U., Babadagli T. (2010). Experimental and visual analysis of co-and counter-current spontaneous imbibition for different viscosity ratios, interfacial tensions, and wettabilities. J. Pet. Sci. Eng..

[9] Sukee A., Nunta T., Haruna M.A., Kalantariasl A., Tangparitkul S. (2022). Influence of sequential changes in the crude oil-water interfacial tension on spontaneous imbibition in oil-wet sandstone. J. Pet. Sci. Eng..

[10] Darvishi H., Goudarznia I., Esmaeilzadeh F. (2010). Effects of rock permeability on capillary imbibition oil recovery from carbonate cores. Trans. C Chem. Chem. Eng..

[11] Strand S., Puntervold T., Austad T. (2008). Effect of temperature on enhanced oil recovery from mixed-wet chalk cores by spontaneous imbibition and forced displacement using seawater. Energy Fuels.

[12] Zhao H., Kang W., Yang H., Zhang H., Zhu T., Wang F., Li X., Zhou B., Sarsenbekuly B., Aidarova S. (2020). Imbibition enhancing oil recovery mechanism of the two surfactants. Phys. Fluids.

[13] Wang F., Wang L., Jiao L., Liu Z., Yang K. (2023). Experimental Mechanism for Enhancing Oil Recovery by Spontaneous Imbibition with Surfactants in a Tight Sandstone Oil Reservoir. Energy Fuels.

[14] Wang Y., Xu H., Yu W., Bai B., Song X., Zhang J. (2011). Surfactant induced reservoir wettability alteration: Recent theoretical and experimental advances in enhanced oil recovery. Pet. Sci..

[15] Pal N., Saxena N., Laxmi K.D., Mandal A. (2018). Interfacial behaviour, wettability alteration and emulsification characteristics of a novel surfactant: Implications for enhanced oil recovery. Chem. Eng. Sci..

[16] Haghighi O.M., Zargar G., Khaksar Manshad A., Ali M., Takassi M.A., Ali J.A., Keshavarz A. (2020). Effect of environment-friendly non-ionic surfactant on interfacial tension reduction and wettability alteration; implications for enhanced oil recovery. Energies.

[17] Meng Q., Liu H., Wang J. (2017). A critical review on fundamental mechanisms of spontaneous imbibition and the impact of boundary condition, fluid viscosity and wettability. Adv. Geo-Energy Res..

[18] Meng M., Ge H., Ji W., Shen Y., Su S. (2015). Monitor the process of shale spontaneous imbibition in co-current and counter-current displacing gas by using low field nuclear magnetic resonance method. J. Nat. Gas Sci. Eng..

[19] Chakraborty N., Karpyn Z., Liu S., Yoon H. (2017). Permeability evolution of shale during spontaneous imbibition. J. Nat. Gas Sci. Eng..

[20] Zhou Z., Abass H., Li X., Bearinger D., Frank W. (2016). Mechanisms of imbibition during hydraulic fracturing in shale formations. J. Pet. Sci. Eng..

[21] Jing W., Huiqing L., Genbao Q., Yongcan P., Yang G. (2019). Investigations on spontaneous imbibition and the influencing factors in tight oil reservoirs. Fuel.

[22] Chang Q., Huang L., Wu X. (2023). A Molecular Dynamics Study on Low-Pressure Carbon Dioxide in the Water/Oil Interface for Enhanced Oil Recovery. SPE J..

[23] Goodarzi F., Zendehboudi S. (2019). Effects of salt and surfactant on interfacial characteristics of water/oil systems: Molecular dynamic simulations and dissipative particle dynamics. Ind. Eng. Chem. Res..

[24] Boldon L., Laliberte F., Liu L. (2015). Review of the fundamental theories behind small angle X-ray scattering, molecular dynamics simulations, and relevant integrated application. Nano Rev..

[25] Wang X., Xiao S., Zhang Z., He J. (2017). Effect of nanoparticles on spontaneous imbibition of water into ultraconfined reservoir capillary by molecular dynamics simulation. Energies.

[26] Yang S., Dehghanpour H., Binazadeh M., Dong P. (2017). A molecular dynamics explanation for fast imbibition of oil in organic tight rocks. Fuel.

[27] Karna N.K., Oyarzua E., Walther J.H., Zambrano H.A. (2016). Effect of the meniscus contact angle during early regimes of spontaneous imbibition in nanochannels. Phys. Chem. Chem. Phys..

[28] Sang Q., Zhao X.-Y., Liu H.-M., Dong M.-Z. (2022). Analysis of imbibition of n-alkanes in kerogen slits by molecular dynamics simulation for characterization of shale oil rocks. Pet. Sci..

[29] Wang S., Wang J., Liu H., Liu F. (2021). Impacts of polar molecules of crude oil on spontaneous imbibition in calcite nanoslit: A molecular dynamics simulation study. Energy Fuels.

[30] Shun W., Jing W., Huiqing L. Forced Imbibition to Enhanced Tight/Shale Oil Recovery: A Molecular Dynamics Simulation Study. Proceedings of the International Conference on Applied Energy.

[31] Kang W.-L., Zhou B.-B., Issakhov M., Gabdullin M. (2022). Advances in enhanced oil recovery technologies for low permeability reservoirs. Pet. Sci..

[32] Wei B., Gao K., Song T., Zhang X., Pu W., Xu X., Wood C. (2020). Nuclear-magnetic-resonance monitoring of mass exchange in a low-permeability matrix/fracture model during CO_2_ cyclic injection: A mechanistic study. SPE J..

[33] Tu J., Sheng J.J. (2020). Experimental and numerical study of surfactant solution spontaneous imbibition in shale oil reservoirs. J. Taiwan Inst. Chem. Eng..

[34] Wang S., Javadpour F., Feng Q. (2016). Molecular dynamics simulations of oil transport through inorganic nanopores in shale. Fuel.

[35] Yuan L., Zhang Y., Liu S., Zhang Y., Chen C., Song Y. (2024). Study on the slip behavior of CO_2_-crude oil on nanopore surfaces with different wettability. Int. J. Heat Mass Transf..

[36] Sui H., Zhang F., Wang Z., Wang D., Wang Y. (2020). Molecular simulations of oil adsorption and transport behavior in inorganic shale. J. Mol. Liq..

[37] Fang T., Wang M., Gao Y., Zhang Y., Yan Y., Zhang J. (2019). Enhanced oil recovery with CO_2_/N_2_ slug in low permeability reservoir: Molecular dynamics simulation. Chem. Eng. Sci..

[38] Zhang C., Dai H., Lu P., Wu L., Zhou B., Yu C. (2019). Molecular dynamics simulation of distribution and diffusion behaviour of oil–water interfaces. Molecules.

[39] Wang Z., Yu C., Zhao J., Guo P., Liu H. (2022). Molecular dynamics simulation for quantitative characterization of wettability transition on silica surface. J. Mater. Res. Technol..

[40] Li G., Su Y., Guo Y., Hao Y., Li L. (2021). Frontier enhanced oil recovery (EOR) research on the application of imbibition techniques in high-pressure forced soaking of hydraulically fractured shale oil reservoirs. Geofluids.

[41] Dong H., Zhu Q., Wang L., Yue X., Fang H., Wang Z., Liu S., Wei S., Lu X. (2023). Effects of Shale Pore Size and Connectivity on scCO_2_ Enhanced Oil Recovery: A Molecular Dynamics Simulation Investigation. Langmuir.

[42] Shi P., Luo H., Tan X., Lu Y., Zhang H., Yang X. (2022). Molecular dynamics simulation study of adsorption of anionic–nonionic surfactants at oil/water interfaces. RSC Adv..

[43] Xu G., Shi Y., Jiang Y., Jia C., Gao Y., Han X., Zeng X. (2018). Characteristics and influencing factors for forced imbibition in tight sandstone based on low-field nuclear magnetic resonance measurements. Energy Fuels.

[44] Albers B. (2014). Modeling the hysteretic behavior of the capillary pressure in partially saturated porous media: A review. Acta Mech..

[45] Wu P., Nikolov A.D., Wasan D.T. (2017). Capillary rise: Validity of the dynamic contact angle models. Langmuir.

[46] Zhang L., Ping J., Tang B., Kang L., Imani G., Yang Y., Sun H., Zhong J., Yao J., Fan D. (2023). Mathematical Model of Two-Phase Spontaneous Imbibition with Dynamic Contact Angle. Transp. Porous Media.

[47] Martic G., Gentner F., Seveno D., Coulon D., De Coninck J., Blake T. (2002). A molecular dynamics simulation of capillary imbibition. Langmuir.

[48] Sun E.W.-H., Bourg I.C. (2020). Molecular dynamics simulations of mineral surface wettability by water versus CO_2_: Thin films, contact angles, and capillary pressure in a silica nanopore. J. Phys. Chem. C.

[49] Wu P., Nikolov A.D., Wasan D.T. (2018). Two-phase displacement dynamics in capillaries-nanofluid reduces the frictional coefficient. J. Colloid Interface Sci..

[50] Rehfeld S.J. (1967). Adsorption of sodium dodecyl sulfate at various hydrocarbon-water interfaces. J. Phys. Chem..

[51] Caineng Z., Zhi Y., Jingwei C., Rukai Z., Lianhua H., Shizhen T., Xuanjun Y., Songtao W., Senhu L., Lan W. (2013). Formation mechanism, geological characteristics and development strategy of nonmarine shale oil in China. Pet. Explor. Dev..

[52] Stevens S.H., Moodhe K.D., Kuuskraa V.A. China Shale Gas and Shale Oil Resource Evaluation and Technical Challenges. Proceedings of the SPE Asia Pacific Oil and Gas Conference and Exhibition.

[53] Zou C., Dong D., Wang S., Li J., Li X., Wang Y., Li D., Cheng K. (2010). Geological characteristics and resource potential of shale gas in China. Pet. Explor. Dev..

[54] Zhu D.-Y., Zhao Y.-H., Zhang H.-J., Zhao Q., Shi C.-Y., Qin J.-H., Su Z.-H., Wang G.-Q., Liu Y., Hou J.-R. (2022). Combined imbibition system with black nanosheet and low-salinity water for improving oil recovery in tight sandstone reservoirs. Pet. Sci..

[55] Wei B., Zhang X., Wu R., Zou P., Gao K., Xu X., Pu W., Wood C. (2019). Pore-scale monitoring of CO_2_ and N_2_ flooding processes in a tight formation under reservoir conditions using nuclear magnetic resonance (NMR): A case study. Fuel.

[56] Fei W., Yingqi R., Qiaoyun C., Zhang S. (2021). A pressure drop model of post-fracturing shut-in considering the effect of fracturing-fluid imbibition and oil replacement. Pet. Explor. Dev..

[57] Lu M., Cao H., Sun W., Yan X., Yang Z., Xu Y., Wang Z., Ouyang M. (2019). Quantitative prediction of seismic rock physics of hybrid tight oil reservoirs of the Permian Lucaogou Formation, Junggar Basin, Northwest China. J. Asian Earth Sci..

[58] Pan L., Dai F., Li G., Liu S. (2015). A TGA/DTA-MS investigation to the influence of process conditions on the pyrolysis of Jimsar oil shale. Energy.

[59] Dong X., Xu W., Liu H., Chen Z., Lu N., Wang W. (2023). On the replacement behavior of CO_2_ in nanopores of shale oil reservoirs: Insights from wettability tests and molecular dynamics simulations. Geoenergy Sci. Eng..

[60] Huang P., Shen L., Gan Y., Maggi F., El-Zein A., Pan Z. (2019). Atomistic study of dynamic contact angles in CO_2_–water–silica system. Langmuir.

[61] Le T.T.B., Striolo A., Gautam S.S., Cole D.R. (2017). Propane–water mixtures confined within cylindrical silica nanopores: Structural and dynamical properties probed by molecular dynamics. Langmuir.

[62] Xu J., Zhan S., Wang W., Su Y., Wang H. (2022). Molecular dynamics simulations of two-phase flow of n-alkanes with water in quartz nanopores. Chem. Eng. J..

[63] Liu L., Ye Z., Lai N. (2022). Characterization Method for Non-uniform Wettability of Shale Oil Reservoir. Int. Core J. Eng..

[64] Koretsky C.M., Sverjensky D.A., Sahai N. (1998). A model of surface site types on oxide and silicate minerals based on crystal chemistry; implications for site types and densities, multi-site adsorption, surface infrared spectroscopy, and dissolution kinetics. Am. J. Sci..

[65] Su Y., Zha M., Ding X., Qu J., Wang X., Yang C., Iglauer S. (2018). Pore type and pore size distribution of tight reservoirs in the Permian Lucaogou Formation of the Jimsar Sag, Junggar Basin, NW China. Mar. Pet. Geol..

[66] Jin J., Yang Z., Chen X., Li L., Yang H., Ju Y., Qiao P., Sun Y. (2021). Characteristics of Micro/Nano Pores and Hydrocarbon Accumulation in a Continental Shale Oil Reservoir—A Case Study of the Lucaogou Formation in the Jimsar Sag, Junggar Basin, Northwest China. J. Nanosci. Nanotechnol..

[67] Wang X., Song Y., Guo X., Chang Q., Kong Y., Zheng M., Qin Z., Yang X. (2022). Pore-throat structure characteristics of tight reservoirs of the Middle Permian Lucaogou formation in the Jimsar Sag, Junggar Basin, northwest China. J. Pet. Sci. Eng..

[68] Cygan R.T., Liang J.-J., Kalinichev A.G. (2004). Molecular models of hydroxide, oxyhydroxide, and clay phases and the development of a general force field. J. Phys. Chem. B.

[69] Nie X., Chen J., Sheng N., Zeng L., Yang H., Wang C. (2017). Effect of water molecules on nanoscale wetting behaviour of molecular ethanol on hydroxylated SiO_2_. Mol. Simul..

[70] Hong X., Yu H., Xu H., Wang X., Jin X., Wu H., Wang F. (2022). Competitive adsorption of asphaltene and n-heptane on quartz surfaces and its effect on crude oil transport through nanopores. J. Mol. Liq..

[71] Zhan S., Su Y., Jin Z., Wang W., Li L. (2020). Effect of water film on oil flow in quartz nanopores from molecular perspectives. Fuel.

[72] Deng Y., Wu Q., Li Z., Huang X., Rao S., Liang Y., Lu H. (2022). Crystal face dependent wettability of α-quartz: Elucidation by time-of-flight secondary ion mass spectrometry techniques combined with molecular dynamics. J. Colloid Interface Sci..

[73] Mayo S.L., Olafson B.D., Goddard W.A. (1990). DREIDING: A generic force field for molecular simulations. J. Phys. Chem..

[74] Jorgensen W.L., Maxwell D.S., Tirado-Rives J. (1996). Development and testing of the OPLS all-atom force field on conformational energetics and properties of organic liquids. J. Am. Chem. Soc..

[75] Wang S., Feng Q., Javadpour F., Xia T., Li Z. (2015). Oil adsorption in shale nanopores and its effect on recoverable oil-in-place. Int. J. Coal Geol..

[76] Berendsen H., Grigera J., Straatsma T. (1987). The missing term in effective pair potentials. J. Phys. Chem..

[77] Zhao J., Yao G., Ramisetti S.B., Hammond R.B., Wen D. (2019). Molecular dynamics investigation of substrate wettability alteration and oil transport in a calcite nanopore. Fuel.

[78] Quezada G.R., Jeldres M., Toro N., Robles P., Toledo P.G., Jeldres R.I. (2021). Understanding the flocculation mechanism of quartz and kaolinite with polyacrylamide in seawater: A molecular dynamics approach. Colloids Surf. A Physicochem. Eng. Asp..

[79] Shen X., Bourg I.C. (2021). Molecular dynamics simulations of the colloidal interaction between smectite clay nanoparticles in liquid water. J. Colloid Interface Sci..

[80] Meng J., Li C., Yan S., Zhang S., Zhang H., Wang G., Yang X. (2022). Atomic-level insights into the mechanism of saline-regulated montmorillonite (001)-salt droplet interface wetting: A molecular dynamics study. Appl. Clay Sci..

[81] Plimpton S. (1995). Fast parallel algorithms for short-range molecular dynamics. J. Comput. Phys..

[82] Humphrey W., Dalke A., Schulten K. (1996). VMD: Visual molecular dynamics. J. Mol. Graph..

[83] Guo L., Tang G., Kumar S. (2019). Droplet morphology and mobility on lubricant-impregnated surfaces: A molecular dynamics study. Langmuir.

[84] Chen J., Mi J.-G., Chan K.-Y. (2001). Comparison of different mixing rules for prediction of density and residual internal energy of binary and ternary Lennard–Jones mixtures. Fluid Phase Equilibria.

[85] Wang L., Lyu W., Ji Z., Wang L., Liu S., Fang H., Yue X., Wei S., Liu S., Wang Z. (2022). Molecular Dynamics Insight into the CO_2_ Flooding Mechanism in Wedge-Shaped Pores. Molecules.

[86] Liu B., Liu W., Pan Z., Yu L., Xie Z., Lv G., Zhao P., Chen D., Fang W. (2022). Supercritical CO_2_ breaking through a water bridge and enhancing shale oil recovery: A molecular dynamics simulation study. Energy Fuels.

