# Supplementary material for: Study on the Effects of Wettability and Pressure in Shale Matrix Nanopore Imbibition during Shut-in Process by Molecular Dynamics Simulations
Source: Molecules. 2024 Mar 1;29(5):1112. doi: 10.3390/molecules29051112 (PMC10934049; doi:10.3390/molecules29051112)
Supplement: Supplementary file 1 [file molecules-29-01112-s001.zip › molecules-2834388-supplementary.pdf]

Study on the Effects of Wettability and Pressure in Shale Matrix Nanopore Imbibition during Shut-in Process by Molecular Dynamics Simulations

Wen Jiang [1,2,3], Weifeng Lv [2,3,4,*], Ninghong Jia [3,4], Xiaoqing Lu [5], Lu Wang [5], Kai Wang [1,2,3] and Yuhao Mei [1,2,3]

1 College of Engineering Science, University of Chinese Academy of Sciences, Beijing 100049, China
2 Institute of Porous Flow and Fluid Mechanics, University of Chinese Academy of Sciences, Langfang 065007, China
3 Research Institute of Petroleum Exploration & Development, PetroChina, Beijing 100083, China
4 State Key Laboratory of Enhanced Oil and Gas Recovery, Beijing 100083, China
5 School of Materials Science and Engineering, China University of Petroleum, Qingdao 266580, China
* Correspondence: lweifeng@petrochina.com.cn

CONTENTS

Figure S1. 1
Figure S2. 2
Figure S3. 2
Figure S4. 3
Figure S5. 3
Figure S6. 4
Table S1. 5
Table S2. 5
Table S3. 5
References 7

Figure S1.

The interaction energy [1] between oil and wall during structural optimization of the oil-pore systems of imbibition systems I and II.

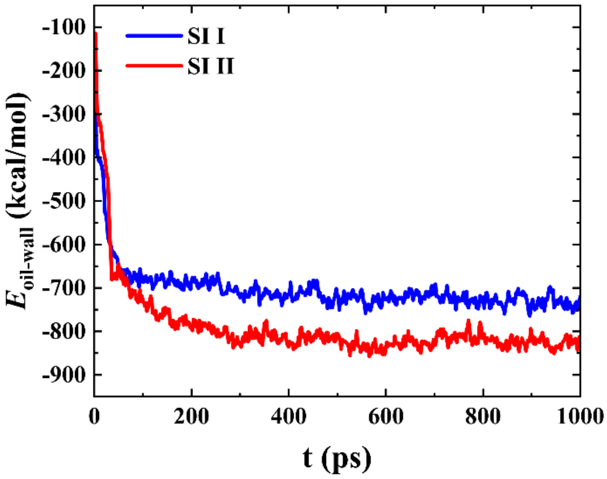

Figure S2.

(a) The interaction energy between water molecules and wall, and (b) hydrogen bond number as a function of time *t* in spontaneous imbibition systems I-II respectively. H-bond exists if the distance of O–O is less than 3.5 Å and simultaneously the angle of H–O···O is less than 30° [2].

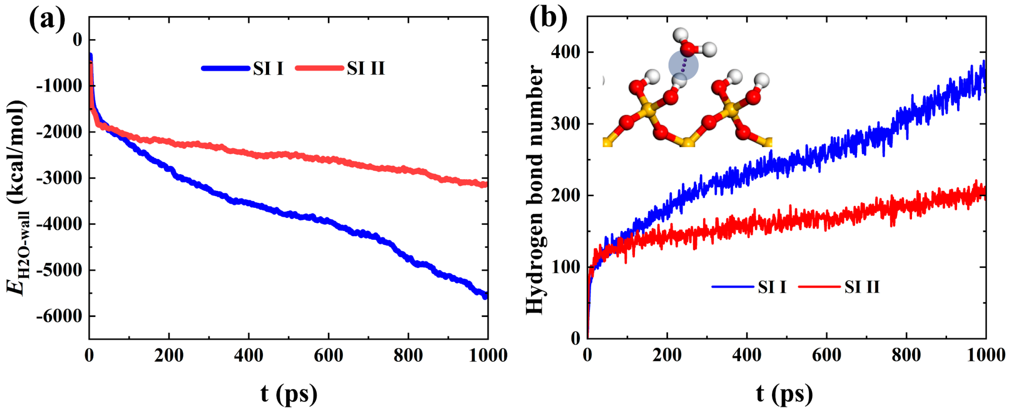

Figure S3.

Molecular model for calculating the interfacial tension of pure oil and water systems.

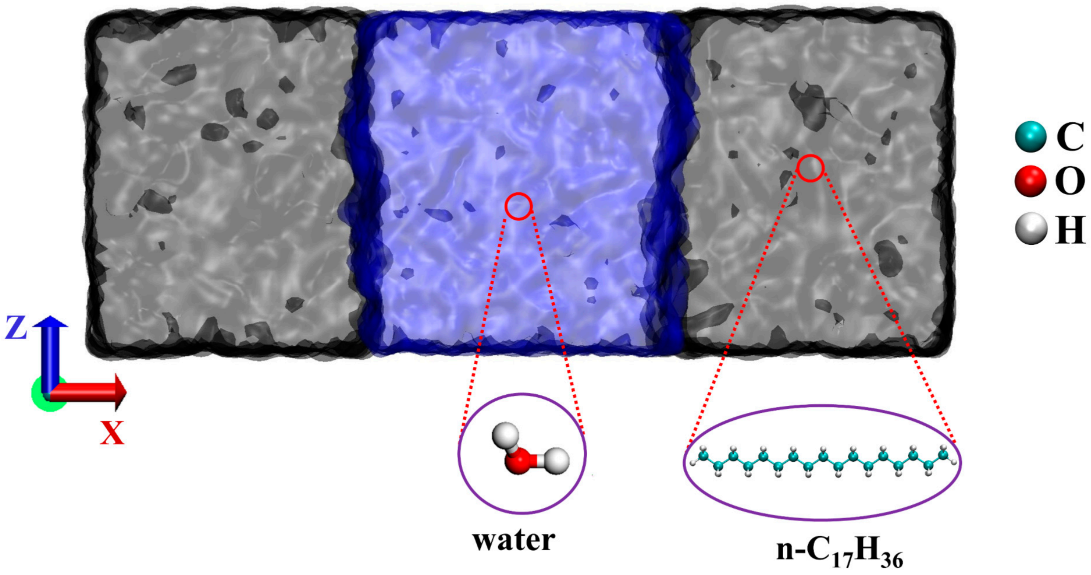

The oil-water interfacial tension is calculated by：

$$\gamma = \frac{L_X}{2}\left(\langle P_{XX}\rangle - \frac{\langle P_{YY}\rangle + \langle P_{ZZ}\rangle}{2}\right)$$

where L_X is the X-direction length of the simulation box, and $\langle P_{\alpha\alpha}\rangle$ (α =x,y,z) is the diagonal element of the pressure tensor, which is averaged over position and time.

Figure S4.

Quartz wall sizes and different modified wall surfaces with different wettability.

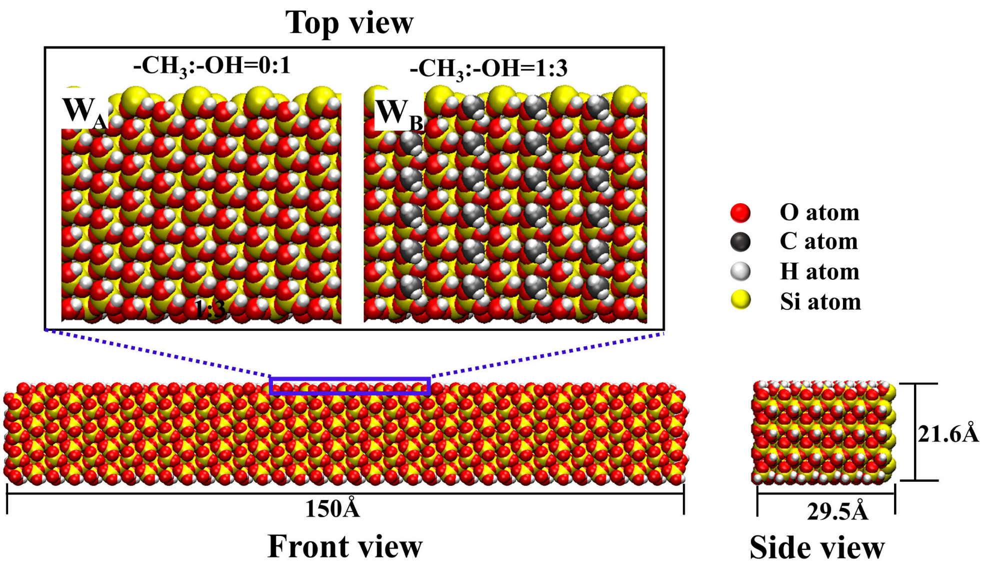

Figure S5.

(a) Initial and (b) equilibrium configuration for static contact angle testing respectively. (c) Schematic diagram of static contact angle calculation method. (d) Static wetting angle of W_A and W_B.

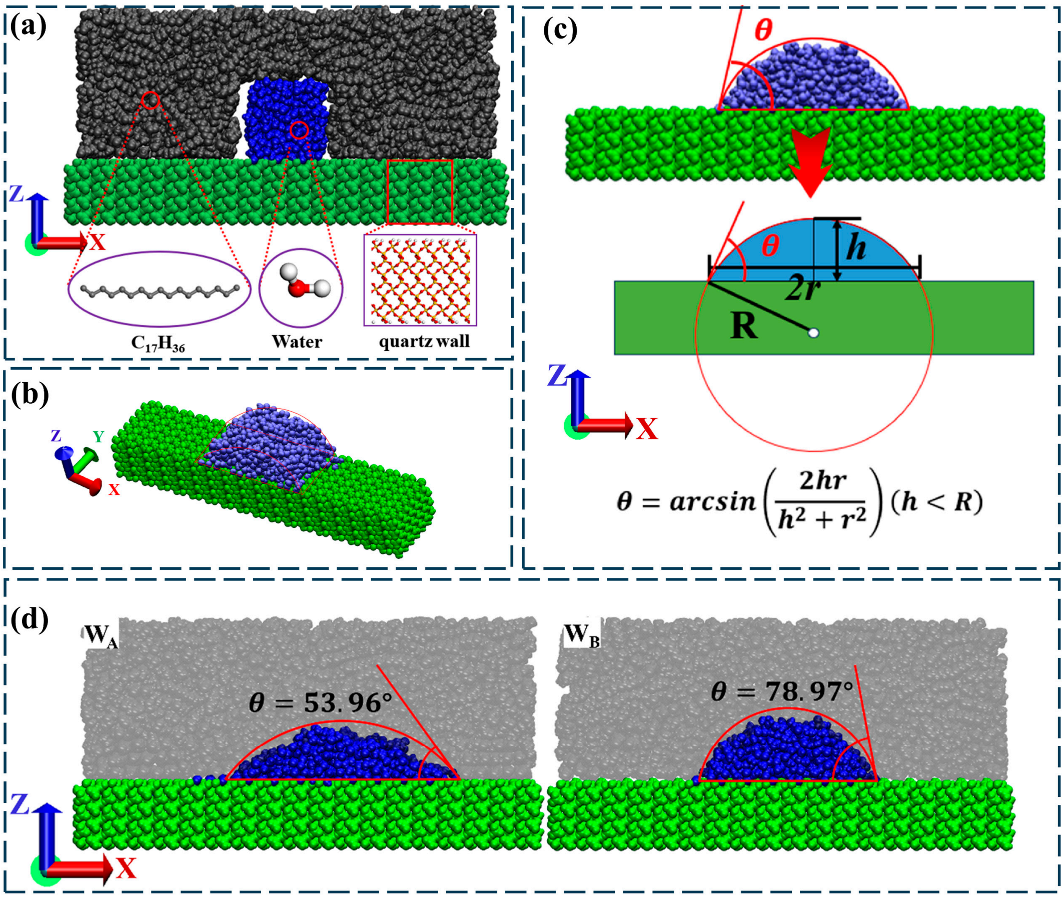

In this work, the static contact angle of the oil-water-quartz wall three-phase system is measured to characterize the different wettability of two modified quartz walls (W_A and W_B). The molecular model for measuring static contact angle is shown in Figure S5a. To ensure the stability and accuracy of the results, referring to the data in Figure S6, shale oil composed of a single component n-$C_{17}H_{36}$ was selected as the wetting external phase, and water clusters were selected as the wetting internal phase. Since the molecular diameter of water molecules is smaller than that of oil molecules, and the edges of water droplets are more “smooth” than the edges of oil droplets, this article adopts the “oil-water droplet-mineral wetting model” that is beneficial to the observation and calculation of contact angles. When using "cube-shaped water clusters" for the wetting model, water spreads in a cylindrical shape on the wall surface, which can eliminate the influence of the three-phase contact line tension on the contact angle. After performing 2ns EMD on this molecular model under reservoir conditions, the stable adsorption configuration of water clusters on the wall surface was obtained as shown in Figure S5b.

Thus, to quantitatively characterize the microscopic contact angles of nano water droplets on different modified quartz surfaces, the water droplets adsorbed on the silica wall surface are approximately regarded as part of an ideal cylinder, as shown in Figure S5c. By geometrically analyzing the bottom surface of the cylinder in the X-Z plane, the contact angle of the oil-water-wall three-phase system can be obtained after stabilization of the adsorption

configuration of the water droplet. The test results are presented in Figure S5d, which matches well with the experimental results of Liu et al. [3].

Figure S6.

The mass fraction of hydrocarbons with different numbers of carbon atoms of (a) JHW05815 Oil Sample, (b) JHW07121 Oil Sample, and (c) J41 Oil Sample.

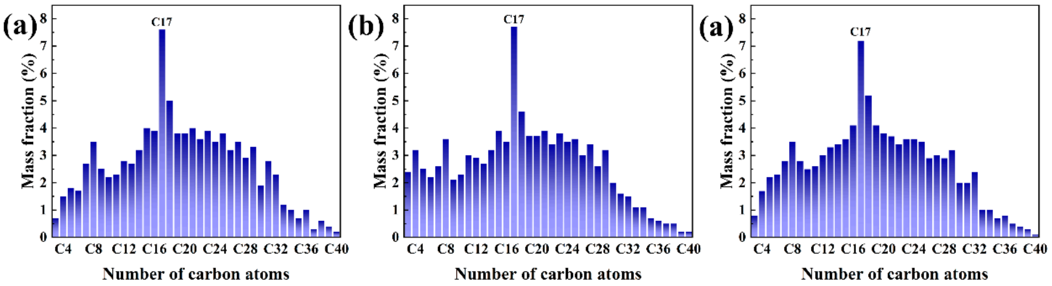

The Xinjiang Research Institute of Petroleum Exploration and Development utilized gas chromatography-mass spectrometry (GC-MS) to analyze shale oil samples from the lower section (P_2l_1) of the Lugaogou Formation. Figure S6 displays the test results of the different hydrocarbon components of the J41, JHW5815, and JHW07121 oil wells, revealing that the carbon number of the hydrocarbon with the highest mass fraction in the chromatographic peak is n-$C_{17}H_{36}$.

Table S1.

The average mass fraction of light, heavy, and medium hydrocarbon components of the Jimsar shale oil.

	J41	JHW05815	JHW07121	Average
Light components(C_3-C_4) (%)	2.6	5.4	2.3	3.43
medium component (C_5-C_{16}) (%)	36.1	34.5	33.3	34.63
heavy component (C_{17}-C_{40}) (%)	61.3	60.1	64.3	61.9

By averaging the data from the oil sample of three wells with different well numbers in Figure S6, the average mass fractions of light (C_3-C_4), medium (C_5-C_{16}), and heavy component (C_{17}-C_{40}) of the Jimsar shale oil were obtained as shown in Table S1 (refer to [4] for the way of dividing the light, medium and heavy components of shale oil). In this MD simulations, the light, medium, and heavy components are replaced by C_4, C_8, and C_{20}, respectively.

Table S2.

The mole fractions and number of each component of the shale oil model

Shale oil components	Mole fraction (%)	Number
n-Butane(n-C_4H_{10})	14.28	110
n-Octane (n-C_8H_{18})	50.12	385
n-Eicosane (n-$C_{20}H_{42}$)	35.6	275

The average mass fraction as shown in Table S1 was converted to a molar fraction to obtain the number of each hydrocarbon component of the simulated shale oil.

Table S3.

Atom charge and detailed interaction parameter of SiO_2, oil, and water.

Non-bond			
**Atom types**	**ε, Kcal/mol**	**δ, Å**	**q, e**
Si	1.8405×10^{-6}	3.3020	2.1
O (O-Si-O)	0.1554	3.1665	-1.05
O (Si-OH)	0.1554	3.1655	-0.95
H (Si-OH)	0.0000	0.0000	0.425
H (Si-O-CH_3)	0.0152	2.8460	0
C (Si-O-CH_3)	0.0951	3.4730	0.338301
O (Si-O-CH_3)	0.0957	3.0330	-0.863301
H (H-C)	0.0300	2.5000	0.06
C (-CH_2-)	0.0660	3.5000	-0.12
C (CH_3-)	0.0660	3.5000	-0.18
O (H_2O)	0.1554	3.1660	-0.8476
H (H_2O)	0.0000	0.0000	0.4238

Bonds		
**Bond Type**	**K_r, Kcal/(mol·Å^2)**	**r_0, Å**
C-C	268.0000	1.5290
C-H	340.0000	1.0900
O-C (Si-O-CH_3)	350.0000	1.3300
C-H (Si-O-CH_3)	350.0000	1.0900
O-H (Si-OH)	554.1349	1.0000
O-H (H_2O)	554.1349	1.0000

Angles		
**Angle Type**	**K_θ, kcal/(mol·rad^2)**	**θ_0, deg**
C-C-C	58.3500	112.700
C-C-H	37.5000	110.700
H-C-H	33.0000	107.800
O-C-H (Si-O-CH_3)	56.2500	109.471
H-C-H (Si-O-CH_3)	56.2500	109.471
Si-O-H	30.0000	107.490
H-O-H (H_2O)	45.7696	109.470

Dihedrals				
**Dihedral type**	**V_1, kcal/mol**	**V_2, kcal/mol**	**V_3, kcal/mol**	**V_4, kcal/mol**
C-C-C-C	1.7400	-0.1570	0.2790	0.0000
C-C-C-H	0.0000	0.0000	0.3660	0.0000
H-C-C-H	0.0000	0.0000	0.3180	0.0000

References

1. Wang, L.; Lyu, W.; Ji, Z.; Wang, L.; Liu, S.; Fang, H.; Yue, X.; Wei, S.; Liu, S.; Wang, Z., Molecular Dynamics Insight into the CO_2 Flooding Mechanism in Wedge-Shaped Pores. *Molecules* **2022,** 28, 188.
2. Liu, B.; Liu, W.; Pan, Z.; Yu, L.; Xie, Z.; Lv, G.; Zhao, P.; Chen, D.; Fang, W., Supercritical CO_2 breaking through a water bridge and enhancing shale oil recovery: A molecular dynamics simulation study. *Energy & Fuels* **2022,** 36, 7558-7568.
3. Liu, L.; Ye, Z.; Lai, N., Characterization Method for Non-uniform Wettability of Shale Oil Reservoir. *International Core Journal of Engineering* **2022,** 8, 179-186.
4. Dong, X.; Xu, W.; Liu, H.; Chen, Z.; Lu, N.; Wang, W., On the replacement behavior of CO_2 in nanopores of shale oil reservoirs: Insights from wettability tests and molecular dynamics simulations. *Geoenergy Science and Engineering* **2023,** 223, 211528.
